# Shape-directed modification of truncated octahedral to coffin-like cobalt-doped ferrite particles by changing the hydrothermal reaction conditions†

**DOI:** 10.1039/d5ra02233a

**Published:** 2025-07-07

**Authors:** Maria Weißpflog, Dietmar Eberbeck, Birgit Hankiewicz

**Affiliations:** a Institute of Physical Chemistry, University of Hamburg Grindelallee 117 20146 Hamburg Germany maria.weisspflog@uni-hamburg.de birgit.hankiewicz@uni-hamburg.de; b Physikalisch-Technische Bundesanstalt (PTB) Abbestraße 2-12 10587 Berlin Germany

## Abstract

Truncated octahedral cobalt ferrite-based nanoparticles were synthesised using a precursor-derived coprecipitation reaction followed by a hydrothermal step. The nanoparticles were characterised regarding their shape anisotropy and size distribution as a function of reaction parameters such as time, temperature, metal salt concentration, base molarity, pressure, and reactor filling volume. Notably, temperature and molarity serve as critical synthesis factors that reduce the polydispersity of the particles to values below 0.1, which is an exceptionally favourable result compared to aqueous coprecipitation reactions. Furthermore, an increase in filling volume resulted in higher proportions of coffin-like nanoparticles due to alterations in flow velocity. The crystallographic assignment of the nanoparticle facets was analysed using high-resolution transmission electron microscopy, selected area diffraction, and angle-resolved scanning electron microscopy measurements. This analysis revealed that the {222} facet exhibited preferential growth in the coffin-like particles. By elucidating the significant reaction parameters for enhanced shape anisotropy, it is possible to increase the magnetic anisotropy constants by assuming a prolate spheroid structure with uniaxial shape combined with cubic magnetocrystalline anisotropy. Contributions of the strain and surface anisotropy are discussed briefly to ensure a comprehensive overview.

## Abstract 2

Truncated octahedral cobalt ferrite-based nanoparticles were obtained by a less hazardous hydrothermal reaction. An increase in the filling volume resulted in coffin-like particles, which exhibit the highest magnetic shape anisotropy constants.

## Graphical abstract

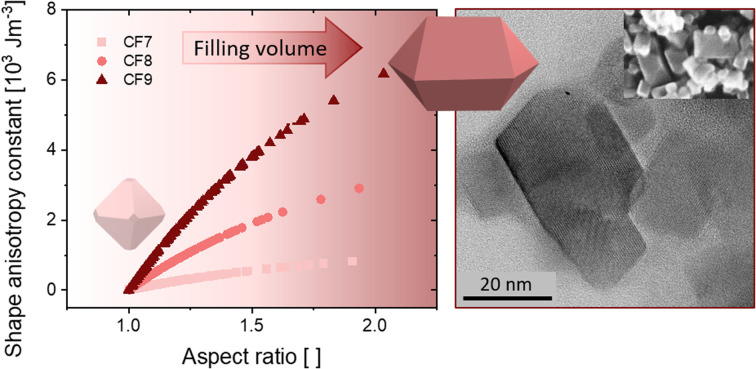

## 1 Introduction

Cobalt ferrite (Co_*x*_Fe_3−*x*_O_4_, CF) is extensively studied for its unique physical, chemical, and magnetic properties, *e.g.*, high chemical stability, remarkable catalytic activity, and large magneto-crystalline anisotropy.^1–4^ Consequently, CF exhibits a wide range of applications as gas sensors,^5^ adsorbent materials for dyes and metal ions,^6–8^ ferrofluid components in electronic devices,^9^ heating elements for magnetic hyperthermia,^10,11^ and particle systems for magnetic imaging and drug delivery.^12,13^ In conjunction with the characteristics of CF within the single-domain regime (critical diameter <100 nm),^14^ dependencies on heating capacity have emerged, especially in the context of medical applications. Notably, the heating capacity of these materials is influenced by the crystallinity, morphology, size, and size distribution of the CF nanoparticles (NPs).^15–17^ Particularly, the magnetic anisotropy (expressed by its constant *K*), which is comprised of surface, shape, magneto-crystalline, and strain contributions, can be altered in this manner.^14,18^ Hence, these parameters affect the Brownian motion as a consequence of thermal fluctuations through viscous drag, as well as the Néel relaxation, which is influenced by the magnetic anisotropy energy barrier.^15,16^ According to Néel's theory, it can be postulated that surface anisotropy becomes negligible for particle diameters exceeding approximately 10 nm.^18^ This is due to the fact that the volume of the particle increases relative to its surface area, resulting in the surface effects becoming relatively insignificant.^19^ Furthermore, Durhuus *et al.* simplified the dependency of *K* on the shape and the magneto-crystalline contributions, resulting in *K* ≠ 0 for elongated and flattened shapes.^17^ It is further assumed that the system consists of single-domain NP exhibiting uniform magnetisation *M* in a liquid suspension at constant viscosity *η*.^20,21^ The NPs were described as prolate spheroids with uniaxial anisotropy.^22,23^ Thus, the representation of the shape anisotropy constant *K*_S_ can be simplified as a function of the aspect ratio AR of the particles.^17^ Due to the dependence of the AR and the associated changes in *K*_S_, it is, therefore, feasible to derive predictions concerning the heating capacity through determinations of the distributions of the particle elongations as predicted by Clarke *et al.*^22^ and McGhie *et al.*^23^ It can be stated that with an increasing anisotropy constant, the average size of the particles decreases at which maximum heat is generated.^24^ However, the size distribution should be considered as well, as magnetic and hyperthermal properties are dependent on the particles' volume and, hence, *K*.^16,25–27^

Numerous approaches, such as decomposition, solvothermal, microemulsion, and template-assisted methods, facilitate the production of shape-anisotropic CF particles in various morphologies, including cubes, stars, octahedrons, nanowires, and plate-like structures.^26,28–33^ However, these techniques often involve toxic precursors or surfactant additives. To achieve shape anisotropy in combination with a green approach, a simple and environmentally friendly method is being developed for this work, enabling future hyperthermia applications. Previous studies have demonstrated that cubic Co_*x*_Fe_3−*x*_O_4_ nanoparticles with a cobalt content of *x* < 0.5 can be synthesised *via* an aqueous co-precipitation method using significantly reduced cobalt salt concentrations, followed by a hydrothermal step.^34^ Parameters, such as reagent concentration and pressure, notably influence the morphology of CF particles. Additional shape-altering factors that arise from the use of a hydrothermal reactor include, *e.g.*, the temperature *T*, the reaction time *t*, the stirring speed *v*, and the filling volume *V*. Variations in *T*, *V*, and *v* lead to significant differences in the flow dynamics between laminar and turbulent flow states, thus affecting the assumed ideal biphasic system composed of particles and solution.^35–37^ Especially, the liquid volume within the reactor serves as a critical factor that influences various aspects of the reaction dynamics.^38,39^ It dictates the mixing efficiency, which is essential for achieving homogeneity and uniform distribution of reactants, as forced convection leads to a more homogeneous reaction.^37^ Furthermore, the filling volume affects the frequency of particle collisions with the reactor walls while keeping the stirring speed constant. The interactions among particles and between particles and walls are crucial for the formation of unique morphologies, resulting in varying particle shapes through processes such as coalescence, fragmentation, and aggregation.^40^ Moreover, the heat transfer efficiency within the solution is closely linked to the filling volume. For example, Ma *et al.* simulated the batch hydrothermal fluid behaviour in dependency on the temperature ranging from 393 K to 473 K. A higher temperature ensures an axisymmetric heat transfer and flow field, resulting in higher velocity and viscous force.^37^ An insufficient liquid volume can lead to localised overheating, which is also connected to an inhomogeneous heating process. Therefore, it is essential to consider the filling volume as a multifaceted parameter that influences both particle morphology and the efficacy of the reaction process. A systematic determination of reactor configuration and characterisation parameters is crucial to receive valuable insights into the optimisation of the reactor synthesis methods with regard to the enhancement of shape anisotropy of the CF nanoparticles.

Additionally, in this work, the aforementioned hydrothermal synthesis for the production of CF was scaled up by a factor of 3. The upscaling of the synthesis and identification of synthesis-altering reactor parameters aim to enhance particle mass yield for future applications while minimising impacts on size distribution. Notably, this discussion is frequently overlooked in fundamental syntheses performed in small autoclaves at scales not exceeding 20 mL of reaction solutions. Given that modifications in reactor dimensions lead to alterations in parameters such as flow velocity and thermal transfer, this discourse is imperative for the advancement of innovative applications.

In this work, the formation of CF particles was mechanistically described in part 1. In part 2, the synthesised particles were examined in terms of their size, size distribution, and shape anisotropy to verify the parameters for a high shape anisotropy combined with a small size distribution. A systematic study was conducted based on reaction and reactor parameters, utilizing techniques such as transmission electron microscopy (TEM) and high-resolution transmission electron microscopy (HR-TEM). The crystallite structure was confirmed by X-ray diffraction (XRD) measurements, and the crystallite sizes as well as strains were obtained using Williamson–Hall (WH) and Halder–Wagner (HW) plots. We want to highlight the investigation of the influence of the filling volume on the particle morphology in part 3, which partially led to coffin-like structures, which were analysed using angle-dependent scanning electron microscopy (SEM) and selected area electron diffraction (SAED) analysis. These particle samples were discussed in part 4 with respect to their anisotropy constants as a function of their shape anisotropy (expressed by the aspect ratio) and size distribution.

## 2 Experimental methods

### 2.1 Materials

All chemicals were used as received without further purification. Iron(iii) chloride hexahydrate (97%), iron(ii) chloride tetrahydrate (97%), and cobalt(ii) chloride hexahydrate (97%) were acquired from Sigma-Aldrich (St. Louis, MO, USA). Disodium hydrogen phosphate dihydrate (99.5%) and sodium hydroxide (99%) were obtained from Merck (Darmstadt, Germany) and Grüssing (Filsum, Germany), respectively. For the stabilisation step, tetramethylammonium hydroxide solution (25 wt%, VWR LLC., Radnor, PA, USA), citric acid (Grüssing, Filsum, Germany), and trisodium citrate dihydrate (Fluka Inc., Buchs, Switzerland) were used. Ultrapure water (Milli-Q quality, resistivity >18.2 MΩ cm) was obtained from a Millipore Milli-Q water purification system from Merck (Darmstadt, Germany). A system manufactured by Berghof Instruments (Eningen unter Achalm, Germany) was used for the hydrothermal treatments. The stainless-steel autoclave features a Teflon liner with internal volumes of 150 mL and adapters to degas the system. The pressure reached a maximum of approximately 5 bar throughout the reaction process. A photograph of the hydrothermal reactor, including the heating block and Teflon inlet, is shown in Fig. S1.†

### 2.2 Preparation of cobalt-doped magnetic particles

An up-scaled protocol for the preparation of the akaganeite precursor solution (2.4 L) was used as described in previous literature, resulting in a nanorod mass concentration of 1.12 wt% in aqueous solution.^34^ For the coprecipitation and hydrothermal step, we utilise the synthesis method demonstrated in prior work.^34^ However, the procedure was scaled up threefold, and reaction parameters like temperature and filling volume were varied. The metal chloride salts of Fe(iii), Fe(ii), and Co(ii) were dissolved in 35.25 mL Milli-Q water and stirred with a magnetic bar (length × width = 30 mm × 8 mm) for around 2 minutes. The precursor solution (27.27 mL solution, 3.39 mmol particle amount, 0.3 g particle mass, respectively) was added to the aqueous metal salt solution and stirred at 1000 rpm for 2 minutes. The solution's final concentration of sodium hydroxide (NaOH) was maintained at 0.5 mol L^−1^. For this purpose, 12.5 mL of a 3 M NaOH solution was injected within 5 seconds using a syringe. By percentage adjustment of the total reaction solution at 25%, 50%, or 100%, the filling volume of the Teflon inlet ranged from ∼17% to 50%. The specific conditions are outlined in Tables S1 and S2.† The hydrothermal reactor was sealed and degassed for 1 minute with nitrogen before being closed through the needle valves at the gas connections. Two distinct temperature profiles were employed for the hydrothermal synthesis. The first profile involved maintaining a temperature of 160 °C for 24 hours. The second profile consisted of a temperature ramp of 120 minutes to achieve 190 °C, followed by isothermal heating at 160 °C for an additional 22 hours. Throughout this thermal treatment, the stirring speed was consistently maintained at 500 rpm. After the reaction, the particles were separated from the solution with a magnet. The particles were washed magnetically three times with Milli-Q water. Subsequently, the nanoparticles were stabilised to obtain negatively charged surfaces according to a process described in Section S1.† Each experiment was conducted once, as the same precursor solution should be used for all experiments to ensure comparability. To address the issue of sample repetition, a statistical analysis of the data and their mean differences was conducted. The significance of these differences was examined at a 5% significance level (*p* = 0.05).

### 2.3 Characterisation methods

#### 2.3.1 High-resolution transmission electron microscopy (HR-TEM)

To resolve the crystal structure and *d*-spacings of the planes, HR-TEM images and SAED patterns were obtained by a double-corrected JEOL JEM 2200FS microscope (acceleration voltage: 200 kV, Cs correctors: Cescor and CETCOR, CEOS). 10 μL of the particle dispersion diluted with 1 mL ultrapure water was dropped on a carbon film-covered copper grid (200 mesh, Science Services GmbH, München, Germany) and dried on air at room temperature.

#### 2.3.2 Scanning electron microscopy (SEM)

Angle-dependent SEM was performed using a Leo Gemini 1550 microscope (Zeiss, Oberkochen, Germany) with an acceleration voltage of 20.00 kV. For sample preparation, the nanoparticle solutions were diluted just as described for TEM preparation, dropped on a silicon wafer, and dried under ambient conditions. A tilt angle was set between the electron beam and the sample holder, which varied between 0°, 25°, 50°, 70°, and 85°.

#### 2.3.3 Transmission electron microscopy (TEM)

The size and morphology of the nanoparticles were characterised using a JEM-1011 microscope (JEOL, Akishima, Japan) at an accelerating voltage of 100 kV. Copper grids, as previously described, were utilised for this purpose. The widths and lengths of one-hundred particles of each sample were measured manually using the open-source software ImageJ (33, Version 1.50i, National Institutes of Health, USA).

#### 2.3.4 Vibrating sample magnetometer (VSM)

Before the VSM measurements, all samples were processed ultrasonically for 1 minute at 90% power to increase the stability. Around 75 μL of the suspension was filled bubble-free into an ULTEM cup and glued onto a perpendicular Quartz sample holder. The mass of the magnetic particles was determined. The quasi-static measurement was conducted using an EZ9 VSM from Microsense LLC (Tempe, AZ, USA). The magnetic characteristics were determined in the magnetic field strength range of ±2.5 T, using the following symmetric full loop protocol: −2.5 T to −1.0 T with steps of 0.5 T, −1.0 T to −0.5 T with steps of 0.1 T, −0.5 T to 0.5 T with steps of 0.025 T, 0.5 T to 1.0 T with steps of 0.1 T, 1.0 T to 2.5 T with steps of 0.5 T, and backwards. The exact mass of the magnetic material was used to express the experimental data in mass moments, as an average of three measurements each.

#### 2.3.5 X-ray diffraction (XRD)

The patterns were obtained using a Philips X'Pert PRO MPD diffractometer (Almelo, The Netherlands) with an X-ray wavelength *λ* of 154.06 pm. Data collection was performed over a 2*θ* range from 10° to 100°. 100 μL of the sample was applied to a silicon wafer and dried under ambient conditions. All patterns were background-corrected and normalised to [0, 1] based on the maximum intensity. The evaluation of the crystal phases was conducted using the Highscore X'pert PRO software from PanAnalytical (Version 2.2.3, Almelo, The Netherlands). The crystallite size *d*_WH_ was determined by WH fits plotting *β* cos(*θ*) *versus* 4 sin(*θ*), where *β* describes the line broadening at half of the maximum intensity of the reflex and *ε* is the intrinsic crystalline strain. Using *k* as a dimensionless shape factor fixed at 0.9 for cubic crystallites, the relationship can be expressed as follows (eqn (1)):^41^1
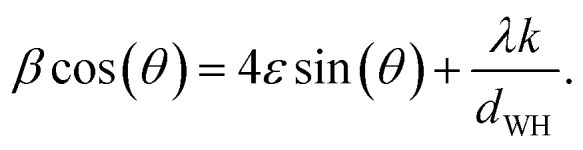


Including the reciprocal lattice point and interplanar spacing, the alternative formula by the HW plot can be adapted, where the crystallite size *d*_HW_ was calculated from the slope and the strain from the intercept (eqn (2)).^41^ The analysis is summarised in Section S2.†2
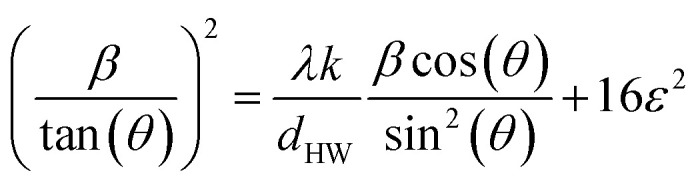


## 3 Results and discussion

### 3.1 Formation of cobalt-doped ferrite particles using akaganeite nanorods as precursors

**Fig. 1:**
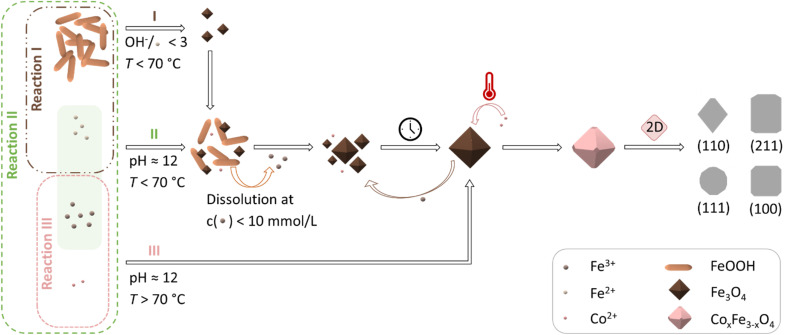
Scheme of the proposed reaction mechanism of the magnetite preparation with Ostwald ripening combined with an ion exchange to obtain cobalt-doped particles. The 2D projections are shown for a truncated octahedron along different crystal surface directions. Reaction I depicts the precipitation of akageneite nanorods with Fe(ii) ions which takes place at Fe(iii) concentrations above 10 mmol L^−1^ under moderate temperatures. Simultaneously, the Massart reaction between Fe(ii) and Fe(iii) hydroxides can proceed to obtain magnetite seeds (reaction II). The resulting decrease in Fe(iii) concentration in the solution leads to the dissolution of the akageneite rods with the release of Fe(iii), which ensures the growth of the nanoparticles. During the process, Co(ii) can be exchanged with Fe(ii) in magnetite, which in turn makes Fe(ii) available again. Fe(iii) can also co-precipitate with Co(ii) at temperatures above 70 °C into cobalt ferrite particles or preferentially form shells around existing magnetite cores (reaction III). In particular, pathway II enables the consistent supply of Fe(iii) and Fe(ii) ions during the growth phase, especially when the akageneite-to-metal salt ratio is set to 0.5.

In this section, the general synthesis route will be described first, followed by an explanation of the particle morphology and an outline of the proposed reaction mechanism illustrated in Fig. 1.

In the initial step of the foundational synthesis of CF1, akaganeite (β-FeOOH) nanorods with lengths of (30 ± 9) nm (*σ* = 30%) by widths of (6 ± 1) nm (*σ* = 17%) were prepared using a hydrolysis mechanism of Fe(iii) chloride at a moderate temperature of 80 °C. The crystalline phase of β-FeOOH was found to be consistent with reference to the Joint Committee on Powder Diffraction Standard (JCPDS) no. 00-034-1266. The analysis of this system was intensively described in previous work.^34,42^ In the second step, Co(ii), Fe(ii), and Fe(iii) chloride salts of a defined ratio to β-FeOOH were filled into the Teflon liner, and the base NaOH was added. The reaction mixture was then processed in the hydrothermal reactor at various parameters (see Section S1†), resulting in non-stoichiometric CF nanoparticles of different morphologies, sizes, and aspect ratios. The crystal phase of the CF particles was confirmed in reference to the JCPDS PDF no. 00-022-1086 for cobalt ferrite, demonstrating a high degree of concordance regarding the diffraction angles and intensities of all reflexes (see Section S2†). As the reaction is performed under a N_2_ atmosphere, hematite and maghemite impurities should be prevented.^43^

To verify the initial mechanism of the coprecipitation at room temperature, TEM and diffraction images of the reaction solutions were taken at 0 s, 60 s, and 300 s after NaOH addition (Fig. S6†), presenting small spheres. Surface tension is the dominant factor influencing the growth of small nanoparticles in the initial phase, as it tends to form spherical shapes, which minimise their surface area and the corresponding surface energy. When the particle size increases to around 20 nm, the shape gradually changes from spherical to cubic, where the surface tension effects and the 〈100〉 direction growth balance. With further increase in diameter, the shape change is due to a higher growth rate along the 〈100〉 directions compared to the 〈111〉 directions, resulting in octahedral particles. This is also due to the lower energy of the {111} surface. This size-dependent morphology behaviour was also demonstrated by López-Ortega *et al.*^44,45^ Additionally, the size and crystallinity of the particles increase with increasing reaction temperature using a hydrothermal step. Fig. S7† illustrates the change of the diffraction rings to dots as well as the spherical to octahedral shape of the particle sample heated to 90 °C after the coprecipitation reaction. Due to the high temperatures and the increased reactivity of the nanoparticle edges, the Ostwald ripening process preferentially occurs at these edges.^46,47^ As a result, rounded octahedral shapes are truncated by the {100} facets, which predominantly appear as rounded rhombuses, rounded rectangles, and spheres in 2D images, as visualised in Fig. 1 (right).

The illustrated reactions in Fig. 1 (left) represent the proposed reaction mechanisms that are likely to occur. First of all, it should be noted that the precursor is needed to form the non-stoichiometric cobalt-ferrite particles as presented in our previous work.^34^ Performing the reaction with the same amount of Fe(iii) chloride instead of precursor nanorods, we obtain mixtures of different particle phases and sizes. This includes goethite rods or stars (length × width ≈ 1000–2000 nm × 200–500 nm), goethite needles (length × width ≈ 1000–2000 nm × 20–100 nm), cobalt ferrite spheres (diameter ≈ 2–6 nm), and/or magnetite cubes (diameter ≈ 15–25 nm or 500–800 nm). Furthermore, the hindering of the akaganeite transformation due to a protective shell around these nanorods leads to the formation of truncated cubic particles instead of truncated octahedra. Performing the reaction without additional Fe(ii) and Fe(iii) salts leads to cobalt oxide and cobalt oxyhydroxide phases, which means that akaganeite cannot be reacted with cobalt chloride under the given reaction conditions to obtain cobalt-doped ferrite particles.

Reaction I depicts the conversion of the nanorods with Fe(ii) hydroxide into magnetite. Due to the phase transformation of akaganeite resulting in magnetite, the elongated form is completely dissolved. Blesa *et al.* reported that β-FeOOH is transformed into Fe_3_O_4_ through dissolution–recrystallisation with hydrazine at pH 9–11.5 and 100 °C under hydrothermal conditions. Even though NaOH is a much stronger base than hydrazine, only dissolution to Fe(OH)_4_^−^ would be observed in the case of NaOH as used in our system.^48^ Ishikawa *et al.* demonstrated in ageing experiments that akaganeite can be converted into magnetite using NaOH in the presence of Fe(ii) at temperatures from 25 °C to 100 °C with an increasing trend.^49^ At room temperature, this phase conversion needs long reaction times of 720 h. However, this process preferentially requires OH^−^/Fe(ii) ratios of 2. At ratios of 2.5, the formation of magnetite takes place from dissolved akaganeite (Fe(OH_4_)^−^) at pH > 11.84 but is reduced or combined with the presence of goethite phases at much higher ratios. As our system is performed at much higher levels of NaOH, the magnetite production will be reduced. The presence of the akaganeite phase after 24 hours of stirring at room temperature confirms this assumption, where NaOH solution was added, but the hydrothermal treatment was not performed (Fig. S6†).^34^

We further assume that the nanorods dissolve at higher temperatures below a certain Fe(iii) ion concentration of 0.01 mol L^−1^.^50^ This is achieved through the consumption of Fe(iii) hydroxides during the formation of octahedral magnetite particles from the coprecipitation reaction of Fe(ii) and Fe(iii) hydroxides (reaction II, Fig. S7a†). At the time, as the nucleation sites are produced from the Massart reaction, the Fe(iii) concentration should be lowered due to the consumption during coprecipitation, reaching the solubility limit of akaganeite. The presence of magnetic material before the hydrothermal step (but also with the presence of the akaganeite phase) indicates that this dissolution process could be reasonable at the used OH^−^/Fe(ii) ratios.^34^ Additionally, the nanorods can act as a heterogeneous surface to form the crystallisation nuclei of magnetite from the Massart reaction. Mao *et al.*^51^ and LaGrow *et al.*^52^ have each resolved the mechanisms of magnetite formation precipitated with the strong base NaOH and the weak base Na_2_CO_3_ (to slow down the precipitation reaction), respectively, with intermediates such as ferrihydrite and goethite or iron hydroxide carbonate [Fe_6_(OH)_12_CO_3_] being formed and promptly agglomerating. In LaGrow's work, amorphous ferrihydrite nanoparticles grow to sizes of up to 2 nm before converting to magnetite and/or maghemite within a period of 3 to 4 minutes. Ferrihydrite acts here as a nucleation seed, while the carbonates serve as feedstock. In contrast, in Mao's study, primary magnetite cores are already present after just 3 seconds using a pH value above 10 (as in our case), which grow to 9.6 nm in size over 2.5 minutes. The growth phase is primarily explained by the agglomeration of periodically occurring intermediates with magnetic cores and magnetite nuclei amongst themselves. The addition of sodium hydroxide in this work ceases after 5 seconds, which should conclude the process of magnetite nuclei formation from Fe(ii) and Fe(iii), as verified by TEM images shown in Fig. S6.† The akaganeite rods would thereafter also be available as ‘feedstocks’ through their dissolution to enable a constant particle growth over time, even at room temperature after 300 s. The parallel integration of Co(ii) ions in exchange for Fe(ii) ions at high temperatures would release Fe(ii) ions that would be available for growth. This allows for the assumption of a constant supply of Fe(iii) and Fe(ii) ions during the growth process, which would lead to a focusing of the size distribution. In some literature, this possibility of continuous provision of precursors or intermediates after the nucleation phase is referred to as an “extended” LaMer process.^53,54^ Furthermore, the Ostwald ripening process would lead to the growth of the already larger particles at the expense of the smaller ones, resulting in a focusing of the size distribution.

It is also noteworthy that Fe(iii) ions and Co(ii) ions have the potential to directly facilitate the formation of cobalt ferrite (reaction III) due to the direct crystal growth mechanism.^55,56^ However, this reaction typically occurs only at temperatures of 70 °C or higher and is not expected at the onset of the reaction at room temperature.^57^ In contrast, Kim *et al.* coprecipitated CF nanoparticles at lower temperatures, showing a clear transition in size and crystallinity between 40 °C and 60 °C.^58^ A mixture of nanocrystalline and crystalline CF phases of 47% and 53%, respectively, was synthesised at a comparable pH value of 12 at moderate temperatures of 60 °C by Thomas *et al.*^59^ This mixture could only be converted into a homogenous sample by calcination at 600 °C, as shown for a sample at pH 11.

Lastly, the hydrothermal step results in the formation of cobalt-doped ferrite through an ion exchange between Fe(ii) ions and Co(ii) ions. This process was also demonstrated by Mitra *et al.*, who showed an ion exchange of Fe(ii) with Co(ii) ions in magnetite nanorods.^60^

The influences of the varied reactor and reaction parameters on the size and size distributions of the obtained particles in comparison to CF1 will be explained based on the models for the preparation of monodisperse particles, as explained by Sugimoto *et al.*^61,62^ Monodisperse nanoparticles with a size distribution coefficient of less than 10% are preferably formed through a three-step process involving initiation, nucleation, and growth, where the nucleation and growth phases are separated. Specifically, a high nucleation rate must be followed by a slow growth phase to achieve this uniformity. During the subsequent growth phase according to LaMer, metal atoms diffuse from the surrounding medium to the nucleation sites, depositing and contributing to particle growth. This growth is thus contingent upon the number of nucleation sites, the concentration of metal atoms, and the diffusion coefficient. The diffusion-controlled Ostwald ripening process would result in an increase in the diameter of the big particles through the dissolution of small nanoparticles. Including most metal oxide growth processes, the mode is mainly reaction-controlled so that the size distribution is sharpened by the initially restricted nucleation phase. Furthermore, the use of a heterogeneous system, where a solid precursor acts as a reservoir, could lead to recrystallization processes during these phases. To establish the actual particle formation mechanism, the reaction kinetics of the mechanisms outlined in Fig. 1 would need to be investigated in a highly time-resolved manner. The mechanistic resolution is also challenging to implement due to the design of the hydrothermal reactors with reaction pressures up to 5 bar, which is difficult to carry out in known setups for, *e.g.*, ultrafast small-angle X-ray scattering measurements in combination with synchrotron X-ray diffraction due to the existing reaction pressure in combination with the high temperatures. However, many studies on iron oxides and ferrites predict the applicability of spontaneous nucleation and/or diffusion-controlled growing processes to their systems, primarily involving thermal decomposition or solvothermal processes.^53,63–65^ Additionally, attention is drawn to the critical examination of the applicability of the LaMer mechanism to various nanoparticle syntheses of, *e.g.*, metal oxides, by Whitehead *et al.*^66^

### 3.2 Influence of the hydrothermal conditions on the morphology

**Fig. 2:**
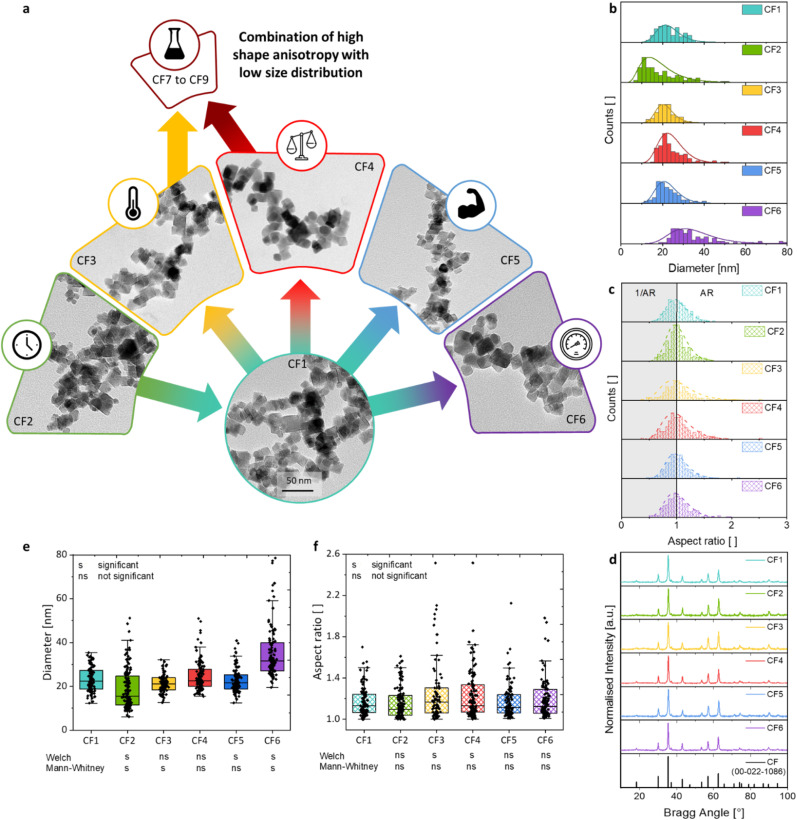
Examination of the synthesis parameters influencing the size, the size distribution, and the shape anisotropy of cobalt-doped particles without changing the composition as well as significance tests of the hydrothermal condition parameters on the size and aspect ratio. (a) An exemplarily TEM image is depicted for the sample CF1 synthesized with an akaganeite to metal salt ratio of 2, a reaction time of 24 hours, a filling volume of 75%, a molarity of the base of 3 mol L^−1^, a reaction temperature of 160 °C, and a pressure of 1 bar. In comparison are shown the samples synthesized with 4 hours reaction time (CF2), a reaction temperature profile of 190 °C for 2 hours and 160 °C for 22 hours (CF3), an akaganeite to metal salt ratio of 1 (CF4), a molarity of the base of 5 mol L^−1^ (CF5), and a pressure of 10 bar (CF6). (b) The diameters *d* of the particle samples are depicted as histograms with the lognormal distribution curves. (c) The aspect ratios (AR) defined as the quotients of widths and lengths are depicted as histograms with the lognormal distribution curve. The histograms were extended by the reciprocal value of AR (grey) to significantly improve the fitting. (d) The composition of the particles was confirmed *via* XRD, indicating no structural changes in the cobalt ferrite reflexes (see reference JCPDS no. 00-022-1086) by changing the reaction parameters. The mean values of (e) the diameters and (f) the aspect ratios of the individual samples were examined for significance in comparison to the reference sample CF1. The significance level was set to 5% (*p* = 0.05). A Welch *t*-test and a Mann–Whitney *U*-test were conducted for each comparison.

The morphologies (Fig. 2a), sizes (Fig. 2b), and aspect ratios (Fig. 2c) of the synthesised particles in dependence on the changed reaction parameters are presented compared to the reference sample CF1. This includes the reaction time *t*, the reaction temperature *T*, the precursor-to-metal salt ratio *r*, the molarity of the base *c*_NAOH_, and the pressure *p*. The size *d*_*i*_ of each particle was obtained from the measured lengths *l*_*i*_ and widths *w*_*i*_ of the particle (*i* = 100), which is shown for samples CF1 to CF6 (see Section S4†). The particle diameter *d*_*i*_ was assessed based on certain assumptions. The first assumption was made that the particles are truncated octahedral, which primarily form rhombuses, squares, and slightly rounded shapes in the 2D projection (Fig. 1). Then, the width of the particles is comparable to the particle images, regardless of the orientation of the crystal surfaces in the 2D projection. The diameter *d*_*i*_ of the measured particles was calculated using eqn (3) based on the assumption that it approximately corresponds to the diagonal of a square with3
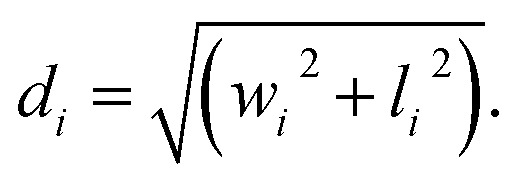


This assumption deviates from rhombuses with an edge angle of less than 90° but applies to the other 2D projections and can be considered as an approximation. The mean size values of *w*, *l*, and *d*, and the corresponding absolute and relative size deviations *σ*, were calculated from the log-normal distribution curves (Section S4†). Based on these size values and the standard deviations, the polydispersity indices (PDI) can be calculated with (*σ*/size)^2^, assuming a Gaussian distribution (Table S3†).

The aspect ratio (AR) of the samples was determined as the mean average value of the individual AR_*i*_ of each measured nanoparticle length and width, where AR_*i*_ was observed through eqn (4) as follows:

4 AR_*i*_ = *l*_*i*_/*w*_*i*_.

The extension of the AR_*i*_ values by their reciprocal values 1/AR_*i*_ (highlighted in grey) leads to Fig. 2c, where a log-normal distribution curve has been fitted to the measured values. This curve is included solely for visual purposes to assess the anisotropy of the particles and was not used for the calculation of the average AR value. A wide, gently declining curve indicates a broad distribution of aspect ratios, suggesting an increased presence of shape-anisotropic particles. Additionally, the representation of the 1/AR_*i*_ values makes it easier to identify any secondary maxima that may occur.

#### 3.2.1 Reference sample

CF1 represents the foundational synthesis upon which all subsequent reactions are based on, modified by altering a single parameter. Possessing a total solution volume of 75 mL (100% solution volume, respectively 50% filling volume), the hydrothermal reaction of CF1 was performed with a precursor-to-metal salt ratio of 2 to 1. A suspension of truncated octahedron nanoparticles with a diameter of (23.3 ± 5.7) nm is obtained after 24 hours of reaction time at 160 °C of reactor temperature (Fig. 2a). By using the temperature profile with a maximum temperature of 160 °C, the target temperature is reached within a maximum of 90 minutes. The size distribution is comparable to aqueous coprecipitation and hydrothermal reactions, with deviations normally ranging from 15% to 30%.

#### 3.2.2 Reaction time

It is first noted that a reaction time of 24 hours is required to achieve a uniform particle suspension with octahedral morphologies. A reduction in reaction time results in sample CF2 with two distinguishable sizes, indicating that the growth phase is not fully completed and Ostwald ripening has not been completed. This time effect is also demonstrated by the distribution of the particle widths, where two maxima can be observed at approximately 8 nm and 18 nm (Fig. 2b). The specification of the overall size distribution yields a higher value of 9.2 nm (∼50%), which also indicates that the reaction is not yet completed.

#### 3.2.3 Reaction temperature

The influence of a higher initial reaction temperature was also investigated, which aimed to enable faster heating of the reactors. For this purpose, a temperature profile was employed, where the mixture was initially heated to 190 °C for 2 hours and subsequently maintained at 160 °C for 22 hours. The increase in the initially maximum *T* influences the heating rate, resulting in comparably smaller nanoparticles with a diameter of 21.4 nm (sample CF3). The size distribution decreases slightly to 4.4 nm, indicating the particle fraction with the smallest size deviation of 21%. It is well-established in the literature that elevated reaction temperatures facilitate the synthesis of nanoparticles characterised by an increased nucleation side number, reduced diameters, and enhanced crystallinity.^58,59,67,68^ Interestingly, the effect of a higher size dispersity or crystallinity cannot be obtained, but the highest average AR of 1.26 and the widest AR distribution (17%) of the CF samples. Therefore, it can be concluded that a higher temperature promotes a higher shape anisotropy of the particles compared with a narrow size distribution within this sample series.

#### 3.2.4 Precursor-to-metal salt ratio

CF4 was obtained by changing the precursor-to-metal salt ratio to 1. Thus, the added metal salt amounts were increased by using the same amount of akaganeite rods in comparison to CF1. This consequently alters the initial monomer concentration, which significantly influences the reactions depicted in Fig. 1 and the underlying mechanistic processes. If CF4 exhibits a higher concentration of metal salts than CF1, it will consequently affect both the nucleation and growth phases. Assuming the same mechanism for the formation of magnetite from the Massart reaction, an increase in the available concentration of metal salts will lead to a faster attainment of the values of saturation and critical concentration. Due to rapid supersaturation and the quick attainment or exceeding of the critical supersaturation concentration, the number of nuclei decreases and the polydispersity increases. Furthermore, the growth phase is influenced by the enhanced availability of free monomers in the solution, which affects the growth potential of smaller particles that would typically have a higher surface energy to grow than larger ones. As a result of this effect of an increased monomer concentration at the end of the NP growth mechanism, larger nanoparticles are formed, especially the crystal size increases. In particular, the shape anisotropy is increased compared to CF1, with a second AR maximum observed around 1.5.

#### 3.2.5 Molarity of the base

Considering that the mechanism comprises coprecipitation followed by a hydrothermal step, the influence of the solution's basicity was primarily examined in the initial phase. For this investigation, NaOH molarities of 1 mol L^−1^ (Fig. S11†) and 5 mol L^−1^ (CF5) were introduced into the reaction mixture, and the resulting size distributions were characterised. The analysis of the diameters and the specification of a size distribution were omitted for the sample precipitated with 1 mol per L NaOH, as it represents a bidisperse sample that would necessitate the separate counting of the small spheres and large cubes, thereby precluding a comparison with the uniform CF samples. The increase in molarity results in a particle size comparable to CF1 or slightly smaller NPs. However, the size distribution is also not significantly altered by the change in molarity from 3 mol L^−1^ to 5 mol L^−1^. Additionally, the average AR is similarly consistent at 1.17. Notably, the crystallite size decreases to (13.9 ± 0.3) nm, and the crystallinity increases to 96%, a characteristic of precipitation methods using higher molarities of the base or initial pH values.^59,69^

#### 3.2.6 Reactor pressure

As depicted in Fig. 2b and S8,† it is clearly observable that the mean size and size distribution significantly enhance with increasing pressure. An increase in the initial pressure in the reactor vessel to 10 bar correlates with a higher slope in the temperature curve compared to experiment CF1. The reaction mixture of CF6 achieves a reactor temperature of 160 °C in 60 minutes instead of 90 minutes, with calculated heating rates of 2.2 K min^−1^ and 1.4 K min^−1^, respectively. The increased pressure results in a substantially heightened size distribution but not a visually higher proportion of shape anisotropic particles with AR < 1.2 (see Fig. S9†). Simultaneously, the mean size of the particles increases considerably. This observation is noteworthy, as it was anticipated that CF6 and CF3 would exhibit similar behaviour, as a higher initial temperature also leads to an increased heating rate. As verified by Talapin *et al.*, the nucleation rate *J*_N_ depends on the activation energy of nucleation Δ*G*^N^.^70^Eqn (5) indicates that this dependency is strongly influenced by the temperature:5
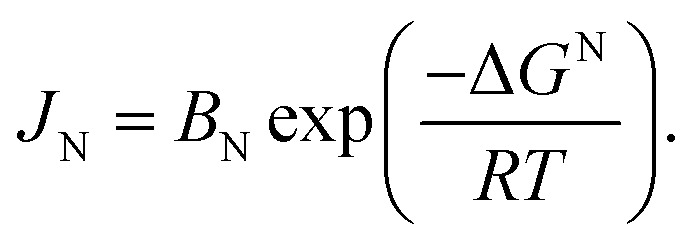


It is demonstrated that an increase in the heating rate affects the NP dispersity and size as the amount of nucleation sites decreases. Therefore, the presence of larger NPs with a higher dispersity is expected with the increase in pressure or heating rate, as demonstrated for sample CF6.

### 3.3 Influence of the hydrothermal conditions on the crystallite size and crystallinity

A brief comparison of the crystallite sizes obtained from the Williamson–Hall (WH) and Halder–Wagner (HW) plots (see Section S2†) with the TEM data is addressed in this section. This alignment further supports the reliability of the crystallite size determinations derived from different analytical techniques. The crystallite sizes exhibit a strong correlation with the widths of the particle samples, especially for the values obtained from the HW plots. The linear fits of HW plot demonstrate notably high coefficients of determination, ranging from *R*^2^ = 0.9945 to 0.9998, which indicates that the HW model nearly completely explains the variability in the variables with respect to the mean. It is well-documented that particle sizes determined through XRD tend to be slightly smaller than those measured *via* TEM.^63,64,71^ This difference is primarily attributed to the presence of crystallite defects, particularly at the surface of the particles, which reduces the measured crystallite size. Furthermore, it is essential to acknowledge that the statistics of the XRD analyses are significantly higher than those of the TEM measurements, which were limited to a sample size of only 100 particles in this study. It should also be noted that the comparison of the *d*_WH_ values of different samples with regard to the absolute particle sizes could be prone to error if the crystallinity is also changed by the change in the respective reaction parameter. Therefore, the crystallinity index (CI) was calculated from the ratio of the integrals of the crystalline peaks and the total area of all peaks (see Table 1 and Fig. S5†).^72^ The relatively sharp reflections indicate a high degree of crystallinity in the samples of 83% to 96% in dependence on the reaction conditions. The change in the crystallinity can result in differences in the crystal sizes, which may vary from the particle sizes observed *via* TEM. However, a similar trend is observed between the TEM data and the XRD results, indicating consistent findings across both methods.

**Table 1:** Summary of the widths and lengths of the synthesised samples CF1 to CF9 and the calculated diameters, as well as the aspect ratios obtained as the mean values of the widths and lengths. The crystallite diameters obtained by the Williamson–Hall and Halder–Wagner plots are determined as well. The crystallinity values were calculated using the quotient of the sum of the area of the crystalline peaks and the total area of all peaks

No.	*w* ± *σ*_*w*_ [nm] ([%])	*l* ± *σ*_*l*_ [nm] ([%])	*d* ± *σ*_*d*_ [nm] ([%])	AR ± *σ*_AR_ [ ] ([%])	*d*_WH_ ± *σ*_*d*,WH_ [nm] ([%])	*d*_HW_ ± *σ*_*d*,HW_ [nm] ([%])	CI [%]
CF1	15.1 ± 3.6 (24)	17.6 ± 4.5 (26)	23.3 ± 5.7 (24)	1.17 ± 0.11 (9)	15.2 ± 0.3 (2)	14.5 ± 0.2 (1)	87
CF2	12.4 ± 6.3 (51)	14.1 ± 6.8 (48)	18.8 ± 9.2 (49)	1.16 ± 0.12 (10)	17.1 ± 0.7 (4)	16.0 ± 0.3 (2)	88
CF3	13.6 ± 3.1 (23)	16.8 ± 4.4 (26)	21.4 ± 4.4 (21)	1.26 ± 0.21 (17)	14.3 ± 0.8 (6)	14.2 ± 0.3 (2)	83
CF4	15.4 ± 3.6 (23)	18.9 ± 5.4 (29)	24.5 ± 6.2 (25)	1.23 ± 0.17 (14)	17.6 ± 0.4 (2)	16.4 ± 0.2 (1)	95
CF5	14.6 ± 3.4 (23)	17.1 ± 4.5 (26)	22.6 ± 5.5 (24)	1.17 ± 0.12 (10)	14.6 ± 0.7 (5)	13.9 ± 0.3 (2)	96
CF6	22.5 ± 6.4 (28)	26.8 ± 8.8 (33)	35.1 ± 10.7 (30)	1.19 ± 0.13 (11)	20.6 ± 2.1 (10)	18.6 ± 0.8 (4)	91
CF7	15.6 ± 3.5 (22)	18.5 ± 4.5 (24)	24.3 ± 5.4 (22)	1.19 ± 0.13 (11)	18.4 ± 1.6 (7)	17.0 ± 0.6 (4)	96
CF8	16.6 ± 4.2 (25)	19.6 ± 5.5 (28)	25.8 ± 6.7 (26)	1.18 ± 0.13 (11)	18.6 ± 1.9 (10)	16.9 ± 0.6 (4)	93
CF9	16.3 ± 3.8 (23)	19.9 ± 5.0 (25)	25.8 ± 6.0 (23)	1.23 ± 0.16 (13)	17.8 ± 0.4 (2)	16.6 ± 0.1 (1)	85

### 3.4 Evaluation of the reaction parameters for a high shape anisotropy combined with a low size distribution

Since the significance of nanoparticle-descriptive parameters is often not assessed, this study will address this aspect (Fig. 2e and f). The considered parameters of this work are the size and aspect ratios, as well as the differences between various particle suspensions. The aim is to provide insights into the challenges of evaluating particle suspensions. The parameters should be identified that do not exhibit a significant change compared to CF1 in terms of the size combined with a low size distribution, while simultaneously showing a significant increase in shape anisotropy. These reaction parameters should then be combined and further studied with alterations in the filling volume. In terms of size and aspect ratios, Welch *t*-tests and Mann–Whitney *U*-tests were conducted.^73–76^ Both methods enable the assessment of the significance of the means of two datasets, which are assumed to be normally distributed in one case and non-normally distributed in the other, respectively. Furthermore, different variances are assumed in both cases, which is often overlooked in most significance tests, yet is considered meaningful when evaluating the size distribution of two different nanoparticle suspensions. A significance level of 0.05 was adopted.

The size distributions indicate that samples CF3 and CF4 do not show a significant difference in means compared to sample CF1 when a normal distribution is assumed. The non-normally distributed testing shows a lack of significance for CF4 and CF5 in comparison to CF1. This implies that the differences in the mean sizes of the samples CF1 and CF3 to CF5 can be regarded as not significant. Consequently, these reaction parameters are suitable if no significant differences in mean sizes are desired. Interestingly, CF3 and CF4 represent the samples that demonstrate a significant difference in aspect ratio compared to the mean of CF1. All other samples, along with changes in the reaction parameters time, base strength, and pressure, exhibit comparable aspect ratios to CF1.

In summary, the following reaction parameters can be identified in comparison to CF1 concerning a narrow particle size distribution combined with a high shape anisotropy (or shape distribution): a high reaction temperature of 190 °C (CF3) and a low molar ratio of precursor to metal salt of 1 (CF4). The temperature was not raised above 190 °C, as this may lead to higher induced strain values and the formation of crystal defects, as demonstrated by the decreased crystallinity and by Fayazzadeh *et al.*^67^ An enhancement in anisotropy may be achieved by further reducing the precursor-to-metal salt ratio.

### 3.5 Coffin-like nanoparticles due to changes in the filling volume

**Fig. 3:**
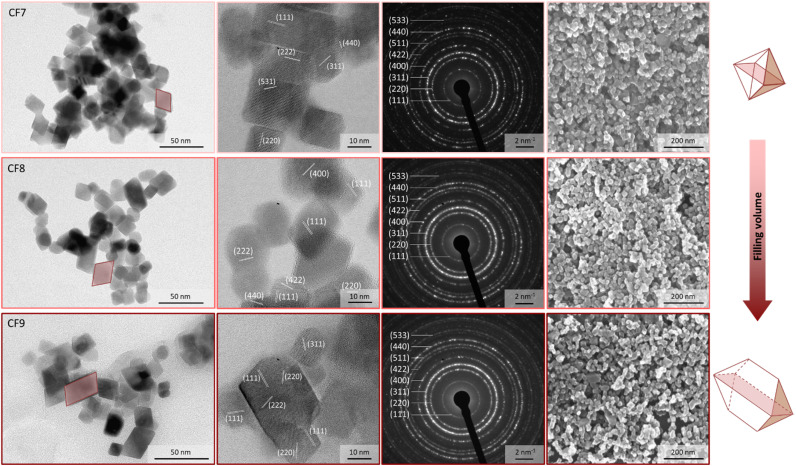
Shape anisotropy and crystal structure of the cobalt-doped particles by variations of the filling volume. The samples were synthesized with a precursor-to-metal salt ratio of 0.5 and a reaction temperature profile of 190 °C for 2 hours and 160 °C for 22 hours. The filling volume varied between 17% (CF7), 25% (CF8), and 50% (CF9) (from above to bottom). For all samples, TEM, HR-TEM, SAED, and SEM images (from left to right) are shown. The 3D structure of the nanoparticle changes from an octahedron to a coffin-like shape.

To enhance the shape anisotropy of the truncated octahedral nanoparticles with narrow size distribution, a controlled reactor reaction strategy was employed based on the findings of Sections 3.2 to 3.4. This involves a low metal salt concentration (ratio of 0.5) and elevated heating rates through higher reaction temperatures. In this context, the influence of the filling volume on the particle morphology was investigated (Fig. 3). The liquid volume within the reactor is a crucial parameter that profoundly influences various aspects of the reaction process. It governs the efficiency of mixing, the frequency of collisions between wall and particle interfaces, inter-particle interactions, and particle-wall interactions, as well as the dynamics of laminar and turbulent flow velocities, and other related parameters such as the heating efficiency of the solution volume. Under a constant heating rate, a reduced volume of solution exhibits a higher temperature increase compared to a larger volume. The observed effect indicates that as the filling volume increases, the time required to reach the target temperature of 190 °C also increases. At 17% of the maximum filling volume (CF7), this target temperature can be attained in approximately 1 hour, likely due to the resulting vapour pressure established in the reactor, a condition not achieved at a fill level of 25% (CF8). After 2 hours of heating, maximum temperatures of 180 °C and 170 °C are reached at filling volumes of 25% (CF8) and 50% (CF9), respectively. When the target temperature is set to 160 °C afterwards, and the power input is subsequently reduced, the system achieves this temperature after 30 minutes for CF7 and after 1 hour for both CF8 and CF9.

The exemplary TEM images of the resulting NP samples are depicted in Fig. 3. In addition to the analysis of the TEM data in histograms (Fig. S12a–d†), box plots were also calculated for the samples CF7 to CF9 (Fig. S12†). The advantage of representing the data as a box plot resides in its independence from the selection of the number and width of the bins, which may influence the histogram representations and the log-normal distribution curves. The diameter of the sample CF9 is increased in comparison to CF1 and CF4 due to the further enhancement of the amounts of metal salts introduced (Table 1). The standard deviation does not change significantly and reaches comparable values of 22% to 26%, similar to those of CF1 and CF4. The polydispersity indices are below 0.07 (Table S4†). This phenomenon is elucidated by the altered mechanism of the initiation phase being shortened and the supersaturation being reached more rapidly, as previously described. In terms of a comparison of the calculated size values obtained as the expected value from a log-normal distribution curve and a boxplot, the mean diameters are ranked in the order of CF7 < CF8 ≈ CF9 in the range of 24.3 to 25.8 nm. Considering the median values, the sizes increase in the sequence of 23.0 nm < 23.5 nm < 23.6 nm, respectively CF7 < CF8 < CF9. Taking both analyses into account, a slight increase in the diameters can thus be observed due to the increase in filling volume. Nevertheless, this increase is not significant due to the variations observed in the TEM analysis. The edge width of the particle samples CF7 to CF9 exhibits no significant differences as well. However, the lengths of the NP show a modest increase. As a result, the mean AR increases from 1.19 to 1.23 for the solution volume of 25% to 100%, corresponding to a percentage deviation of 11 to 13%. These values are comparable with the median aspect ratios for CF7 (1.14), CF8 (1.13), and CF9 (1.17), depicted in Fig. S12e.† The increased AR is likely associated with the pronounced differences in the vertical flow behaviour within the reactor vessel. Differences in flow velocities within the profile arise, which consequently impact the Reynolds number. Thus, the flow regime becomes alterable, categorising it into laminar and turbulent flow. With a higher fill level in a cylindrical vessel, the variance of the stirrer flow perpendicular to the stirrer increases. As a result, the previously mentioned parameters related to the filling volume, such as the number of collisions and the temperature profile, are significantly altered within the reactor. In either case, an increased filling volume results in a rise in the aspect ratios of the particles.

**Fig. 4:**
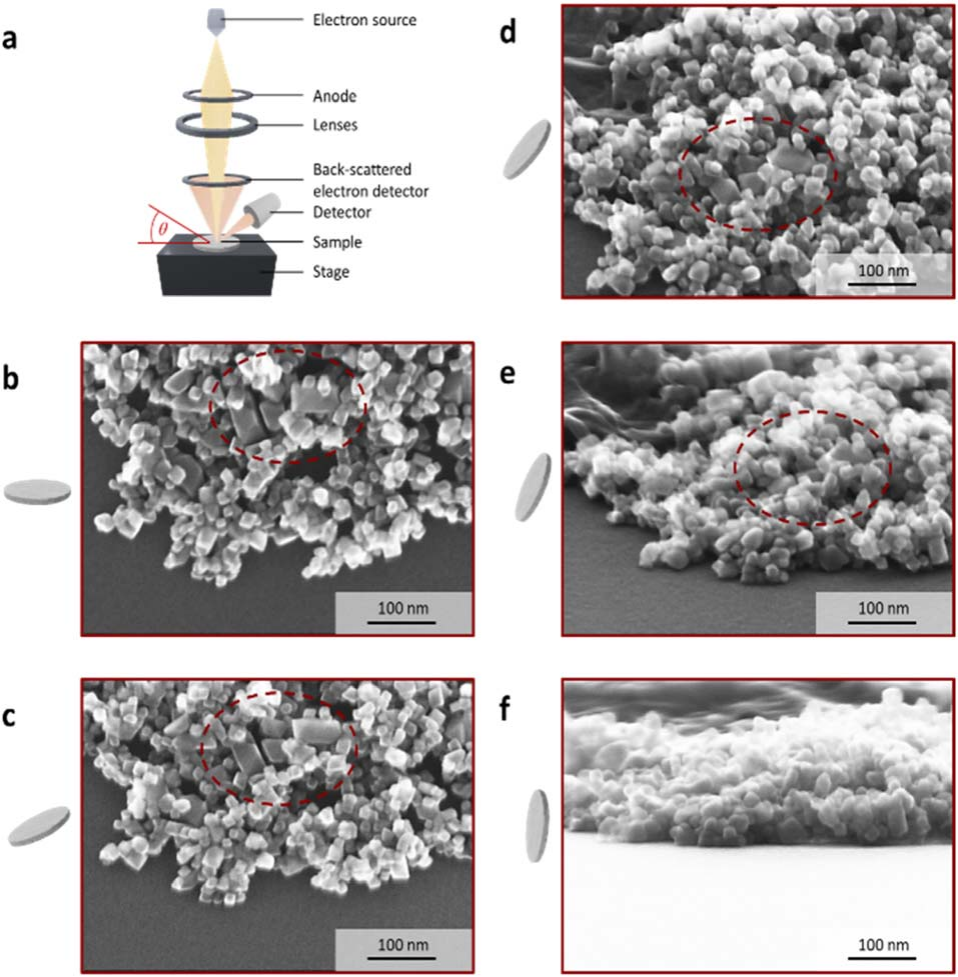
Angle-dependent SEM measurements of the cobalt-doped nanoparticles for a filling volume of 100%. (a) The schematic structure of the SEM is depicted, where the angle *θ* between the stage and the electron source can vary between 0° and 85°. The sample is dropped and dried on a polished silicon wafer, which is glued on the stage. The angle-dependent SEM images of the sample CF9 are presented for angles of (b) 0°, (c) 25°, (d) 50°, (e) 70°, and (f) 85°.

The increase in the aspect ratio can be attributed to a higher proportion of distorted parallelograms (2D representation) observed in sample CF9 (marked area in Fig. 4). HR-TEM images further indicate the formation of elongated honeycomb-like projections. When a foundational octahedral 3D structure is considered (see CF1 and CF7), this truncated octahedron structure appears to undergo additional distortion. The elucidation of the 3D structure was achieved through angle-dependent SEM measurements (Fig. 4 and Section S6†), which demonstrate a pronounced representation of the coffins (highlighted circle) in sample CF9 as a function of the angle, ranging from 0° to 85°. The characterisation of crystal planes in the HR-TEM images, facilitated by inverse fast Fourier transformation (Section S7†), suggests that the preferential growth of the {222} plane is responsible for this elongated distortion. The width of the coffins is defined by the growth of the {111} plane. A visually higher proportion of these elongated octahedral nanoparticles is observed as a result of the increase in the filling volume. This is accompanied by an elevation in the liquid level within the thin Teflon vessel, decreasing the frequency of particle-wall collisions. Additionally, there is an alteration in the flow pattern generated by the stirrer within the reactor vessel, which is anticipated to exert an influence on both the particle morphology and anisotropy. The flow velocity and mixing along the reactor axis diminish with increasing fill height from 25% to 100% while maintaining a constant cross-flow rate. Due to the preferred growth of the {222} crystal facet of the particles, there must be an influence on the particle growth along this specific crystal plane, or alternatively, the other crystal planes must be blocked. Since no crystal-blocking agents are added during synthesis, this effect is likely resulting from the significantly higher variability in flow and temperature profiles within the reactor at higher fill levels. In combination with this, the further increased metal salt concentration in the reactor syntheses from CF7 to CF9 could also influence the growth phase, as more metal ions are available. We therefore hypothesise that the particle growth phase is being affected to obtain coffin-like particles.

The crystallographic analysis of the SAED images enables the assignment of the inverse spinel structure to the NP. The same crystalline phases are determined *via* HR-TEM and SAED analysis. The observation of bright spots in the diffraction pattern indicates the presence of larger crystallites within all samples. This aligns with the particle size distribution data illustrated in Fig. S12.† Furthermore, lattice patterns can be observed in the TEM images of the larger particles, with enhanced evidence in the HR-TEM images for almost all particles depicted. In certain instances, these patterns do not extend to the particle edges, indicating the presence of amorphous regions in these areas. This would result in a reduction of the magneto-crystalline anisotropy, particularly pronounced in CF7. Upon examining the particle morphology of the octahedrons and their shape irregularities, a certain uniaxial anisotropy can be assumed for all samples from CF7 to CF9.^77^

### 3.6 Determination of the magnetic anisotropy constants

**Fig. 5:**
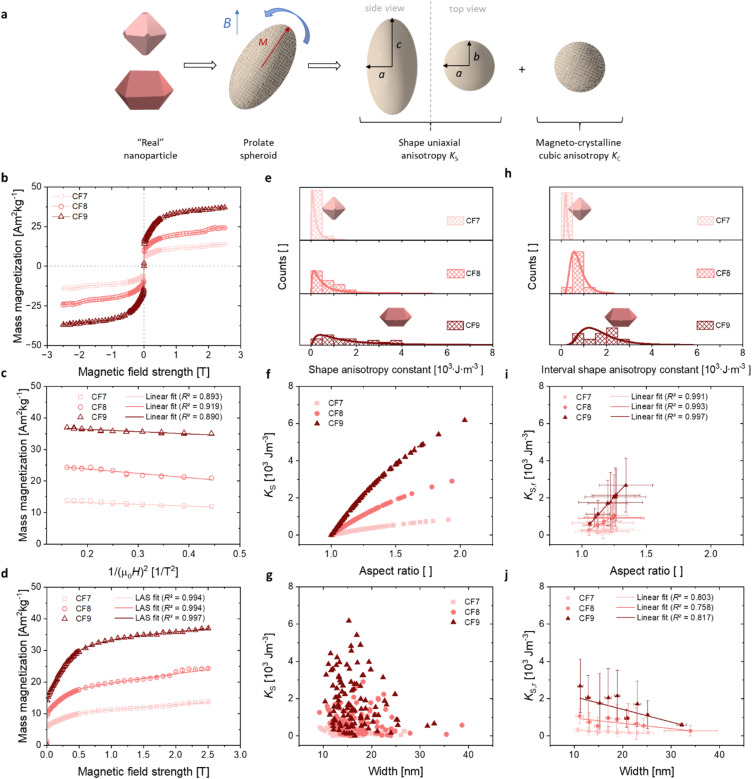
Magneto-crystalline, shape, and interval shape anisotropy constants as a function of the aspect ratios and widths in dependence on the filling volume. (a) Scheme of the illustration of the uniaxial shape and the cubic magneto-crystalline anisotropy contributions using a simplified model of a prolate spheroid to describe the particle anisotropy. (b) The mass-normalised magnetisation curves (related to the mass of the magnetic material) are shown. (c) The high-field region of the *M*(1/*H*^2^) curves are fitted linear to obtain the saturation magnetisation. (d) Using the LAS approach, the magneto-crystalline anisotropy constant can be calculated. (e) The distribution of the shape anisotropy constants *K*_S_ shows a median increase, with a notable rise in the frequency of values above 2.5 × 10^3^ J m^−3^, particularly in the CF9 sample. (f) This enhancement in *K*_S_ can be attributed to the increase in the aspect ratio, which is primarily due to the presence of coffin-like particles. (g) The display of *K*_S_, which varies with width, shows no dependence. (h) A mean value can be calculated from the distribution of the interval shape anisotropy *K*_S,*r*_ which clearly shows a shift of the mean values to higher *K*_S,*r*_. (i) The linear dependency of the AR on *K*_S,*r*_ for different filling volumes are depicted with *R*^2^ ≥ 0.991. (j) The width-dependent illustration indicates an inversely trend to AR.

The assessment of the magnetic anisotropy in this study is especially conducted through the combination of magneto-crystalline and shape anisotropies, respectively, *K*_C_ and *K*_S_. In this context, the approaches of the theoretical and experimental works by Faílde, Clarke, McGhie, and Durhuus *et al.* are applied.^17,22,23,78^ The illustration of the approximations made is depicted in Fig. 5a, where the sum of *K*_C_ and *K*_S_ is considered to obtain the magnetic anisotropy constant *K*. Additionally, an experimental approach for evaluating *K* was determined to enable a comparison with the calculated anisotropy values, which is summarised in Section S8.†

If the magnetisation *M* of a single-domain particle is uniformly distributed above the particle volume *V*_P_, the magnetic energy *E* can be written as

6 *E* = *KV*_P_ sin^2^ *θ*

with *θ* as the angle between the magnetic moment *m* and the easy direction of magnetisation.^14,16,21^ The energy contributions from magneto-crystalline and shape anisotropies correspond to *E*_C_ and *E*_S_, respectively.^14,18^

#### 3.6.1 Magneto-crystalline anisotropy by a modified law of approach to saturation (LAS)

Due to the cubic magneto-crystalline structure of CF, this particulate system exhibits a magnetisation process that deviates from uniaxial magneto-crystalline anisotropy.^22,23^ The existence of six easy directions along the cube edges of the crystal decreases the probability that a particle must rotate about the hard direction in the presence of an applied magnetic field. This alteration results in a reduction of the energy barrier to reversal to *E*_B,c_ = 1/12*K*_C_*V* compared to observation in uniaxial systems.^22,23^ Consequently, the value of the energy barrier *E*_B,c_ is reduced to 8.75 × 10^3^ J m^−3^. Furthermore, the first-order *K*_C_ of 1.05 × 10^5^ J m^−3^ is expected to be reduced due to the presence of non-stoichiometric CF particles, which exhibit a higher iron content with a maximum cobalt-to-iron ratio of 0.2. This is based on the quantities of materials employed for the synthesis, their distribution across the particle volumes, and on determinations from previous studies that identified the range of 0.25 < *x* < 0.45 through atomic emission spectroscopy measurements.^34^ Magnetite possesses a *K*_C_ value of −1.25 × 10^4^ J m^−3^ at room temperature,^79^ which, under the same cubic crystal symmetry, would diminish the energy barrier to reversal to 1/12 of this value. Therefore, |*K*_C_| is anticipated to range between (1.25–10.5) × 10^4^ J m^−3^, respectively an energy barrier *E*_B,c_ of (1.0–8.8) × 10^3^ J m^−3^.^79^ In conclusion to the cobalt-to-iron content in this study, a maximum size- and AR-independent *K*_C_ of 2.1 × 10^4^ J m^−3^ (Table 2) and *E*_B,c_ of 1.8 × 10^3^ J m^−3^ were hypothesised.

**Table 2:** The saturation magnetisation, magnetic domain size, magneto-crystalline, shape, strain, and surface anisotropy constants are evaluated for the samples CF7 to CF9

No.	*M*_S_ [A m^2^ kg^−1^]	*d*_mag_ [nm]	*K*_C,LAS_ [10^4^ J m^−3^]	*K*_S_ [10^3^ J m^−3^]	*K*_S,*r*_ [10^3^ J m^−3^]	*K_ε_* [10^4^ J m^−3^]	*K*_O_ [10^−4^ J m^−2^]
CF7	14.3 ± 0.2	23.4 ± 0.3	3.2 ± 0.4	0.2 ± 0.2	0.2 ± 0.1	−0.6 ± 0.6	1.3 ± 0.2
CF8	26.7 ± 0.4	18.1 ± 0.2	5.9 ± 0.7	0.8 ± 0.6	0.8 ± 0.3	−1.5 ± 0.7	1.8 ± 0.2
CF9	37.8 ± 0.2	16.7 ± 0.1	4.9 ± 1.1	1.9 ± 1.4	1.7 ± 0.8	−1.1 ± 0.2	1.4 ± 0.3

The experimental verification of the magneto-crystalline magnetic anisotropy *K*_C_ was conducted by VSM measurements and plotting the mass magnetisation *M* in A m^2^ kg^−1^*versus* the magnetic field strength *μ*_0_*H* in Tesla (Fig. 5b). There is no significant opening of the hysteresis loops because the particles within the suspension may align along the external field. It is evident that no saturation is reached at high magnetic field strengths due to the slight increase in the curve. Therefore, a modified LAS approach was used, as described in eqn (7):^80,81^7
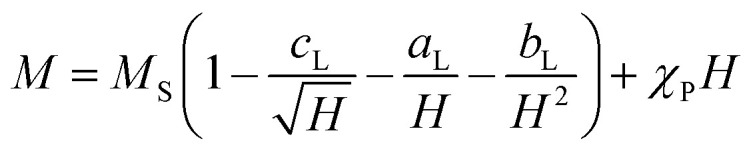
Here, *a*_L_ describes the inhomogeneity parameter, which presents the dislocations in the system. *c*_L_ denotes structural defects and fluctuations, and *χ*_P_ the high field susceptibility. The relationship presented in eqn (7) describes the magnetisation at high field strengths, especially when these are much higher than the coercivity values. We would like to point out that discussions about these assignments of the LAS approach are still ongoing.^82,83^ Compared to the Langevin function, the modified LAS theory takes particle interactions into account.^83^

The saturation region can be described by eqn (8), where the terms with *a*_L_ and *c*_L_ can be neglected due to the linearity shown in Fig. 5c:^80^

8 *M* = *M*_S_(1 − *b*_L_/*H*^2^).

Thus, the saturation magnetisation values *M*_S_ were calculated from the *y*-intercepts by linear fitting of the *M vs.* 1/(*μ*_0_*H*)^2^ representation in the high-field range (Fig. 5c). The *M*_S_ values increase from (14.3 ± 0.2) A m^2^ kg^−1^ to (37.8 ± 0.2) A m^2^ kg^−1^ with the filling volume used. Then, *K*_C,LAS_ was determined by fitting the *M*(*μ*_0_*H*) curves from 0.4 T to 2.5 T (Fig. 5d), using the relationship of *b*_L_ with the magneto-crystalline anisotropy constant described *via*eqn (9):^80^9
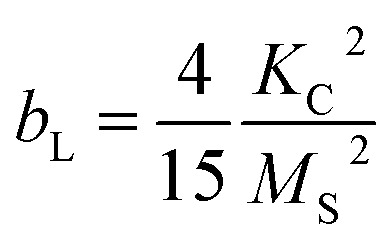


All samples exhibited an *R*^2^ value of >0.994 (Fig. 5d). The *K*_C,LAS_ values of (3.2 ± 0.4) × 10^4^ J m^−3^ to (5.9 ± 0.7) × 10^4^ J m^−3^ fall within the range discussed above. *K*_C,LAS_ follows the trend of the strain (see Fig. S4†). The decreased values compared to CF (*K*_C_ = 1.1 × 10^5^ J m^−3^) can be explained by the lower cobalt content. The fitting, which was performed over a broader range using the LAS method compared to the linear fitting, served as the basis for the *K*_C_ values to enable comparison with the *K* constants. Note that the LAS approach regards the rotation of the vector of the magnetic moment from the direction of the easy axis towards the external field direction.^84^ This means that the particles should be fixed during the measurement. Here, the measurements were performed on suspensions and partially on immobilised magnetic NP. However, the magnetic NP were aggregated and precipitated due to an interaction. Thus, we presume that the MNP rotation is hindered and that the assumption of eqn (8) with eqn (9) is valid. While the alternating current susceptibility (ACS) data show that almost all magnetic NP of CF7 are immobilised, only 65% of CF8 are so (Table S7†). In case of CF9, even more NP appear not fixed and are partially organised in large clusters (Section S8†). Therefore, the calculated values for the crystal anisotropy should be interpreted with caution. Furthermore, due to possible interactions between the magnetic NP, the *K*_C_ values should be interpreted as effective values. Nevertheless, the ratio of the corresponding *K* values between CF7 and CF8 obtained from analysis of the data of ACS (Table S7†) matches the ratio of the related *K*_C_ values obtained from the modified LAS approach (Table 2).

#### 3.6.2 Determination of the shape anisotropy constants *K*_S_ by assuming a prolate spheroid

*K*_S_ of CF7 to CF9 were determined assuming uniaxial shape anisotropy. Gandia *et al.* evaluated this assumption for cubic shape anisotropic particles with an elongation ≥2%, which includes cubic, octahedral, and truncated-octahedral morphologies.^85^ Considering a non-spherical shape, which can be simplistically represented as a prolate ellipsoid with the principal semiaxes *a*, *b*, and *c*, this ellipsoidal shape aligns with their magnetic moments along the longest axis. This orientation represents the direction associated with the lowest energy of shape anisotropy.^86^ Then, the uniaxial shape anisotropy constant *K*_S_ can be expressed as10
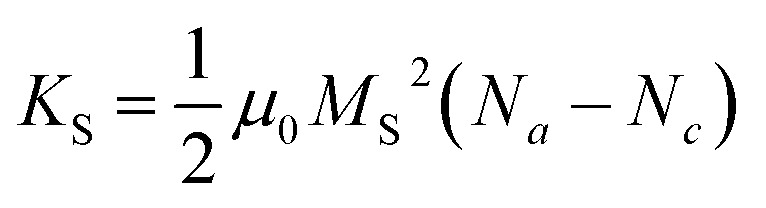
with *μ*_0_ as the vacuum magnetic permeability of 4π × 10^−7^ H m^−1^ and *M*_S_ as the saturation magnetisation in A m^−1^.^16,21,22,87^ Since the magnetisation is significantly influenced by particle size and distribution, and the synthesised samples exhibit a considerable variance in particle widths, the measurement of the average magnetic magnetisation was deemed inappropriate. To facilitate an assessment of the shape anisotropy constants that is independent of magnetisation, *K*_S_ could be expressed as multiples of the reference value *μ*_0_*M*_S_^2^. As this formulation would not facilitate comparisons with the existing literature, which predominantly reports *K*_S_ values in units of J m^−3^ (or emu cm^−3^), *K*_S_ was depicted in dependency on the magnetisation-dependent expression. Therefore, the model was investigated using the experimental *M*_S_ values of the samples CF7 to CF9.

*N*_*a*_ and *N*_*c*_ describe the dimensionless demagnetisation coefficients parallel to the short symmetry axis *a* and along the long symmetry axis *c* represented by11
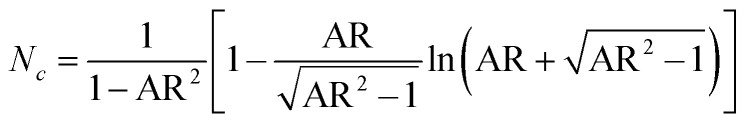
and12
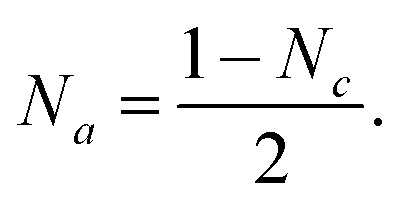


The particle elongation was determined by measuring the long semiaxis *c* and the short semiaxis *a* (Fig. 5a). These values approximately correspond to the measurements obtained from the width and length assessments used to calculate the AR of the octahedrons discussed in this work.

All *K*_S_ values were then plotted similarly to the size distribution histograms (Fig. 5e). The log-normal distribution curves are only inserted for visual orientation. A determination of *K*_S_ with the help of the lognormal distribution function is not meaningful, as the error values exceed the mean values. The average values and standard deviations of normal distributions were calculated, which are presented in Table 2. The average values follow the trend CF7 < CF8 < CF9, which is consistent with the observed AR. It is evident that the calculation of the mean provides limited information, as it likewise exhibits large error values. However, it is obtained that the distribution of the *K*_S_ values in sample CF9 exhibits a notably higher variability compared to the other samples, indicating a broader dispersion of the data points. A significant second increase in frequencies is observed in sample CF9 beginning at a *K*_S_ of 2.5 × 10^3^ J m^−3^, which corresponds to an AR > 1.25. We assume that the higher variance and the second maxima in the histogram can be attributed to the higher variance in the flow and temperature profiles during the synthesis, resulting in a larger proportion of shape-anisotropic particles.

The visualisation of eqn (10) in Fig. 5f depicts the dependence of *K*_S_ on AR, revealing a marked increase in *K*_S_ with increasing AR. From eqn (10)–(12), it is evident that for an AR = 1 (*i.e.*, a sphere or symmetric octahedron), *N*_*c*_ equals 1/3, leading to *K*_S_ = 0. In contrast, the curve should approach *K*_S,max_ at 0.25*μ*_0_*M*_S_^2^ as the AR receives infinite values. This results in approximately 1.3 × 10^4^ J m^−3^ for sample CF9 with the highest *M*_S_, and in values of 6.3 × 10^3^ J m^−3^ and 1.8 × 10^3^ J m^−3^ for CF8 and CF7. The obtained maxima of *K*_S_ in this work are 0.8, 2.9, and 6.2 × 10^3^ J m^−3^ for CF7, CF8, and CF9, respectively. The highest value of *K*_S_ corresponds to an AR of approximately 2.0, observed in the CF9 sample. Although no clear correlation with the mean sizes obtained *via* TEM is discernible (Fig. 5g), only the previously mentioned broader distribution of the sizes can be observed. As we cannot obtain a cross-section between *K*_S_ and *K*_C_ in both diagrams, the magneto-crystalline anisotropy is expected to dominate for all AR and sizes.

To assess the impact of the particle size distribution on AR and *K*_S_, the sizes used for calculations of the shape anisotropy and the aspect ratios were divided into ranges corresponding to a width interval of 2 nm (indexed by “*r*”). A linear increasing trend from diameter to width is clearly observable (Fig. S19a†). In contrast, the AR_*r*_ values exhibit a decreasing trend in dependence on the widths (Fig. 5j) as the AR_*i*_ for each particle size decreases with the width of the particles for each filling volume (Fig. S19b†). The distribution of the *K*_S,*r*_ values is shown in the histograms in Fig. 5h. In contrast to the distribution in Fig. 5e, the mean values can be determined using the logarithmic distribution curve in the interval representation, which are presented in Table 2. The shift in the mean values with the filling volume is clearly noticeable. Additionally, a more pronounced second peak in the distribution can be observed for sample CF9, located at approximately 2.5 × 10^3^ J m^−3^. This corresponds to an AR > 1.3, as determined from the AR-dependent representation of the data in Fig. 5i. *K*_S,*r*_ demonstrates an increasing trend with AR_*r*_ in the interval representation with a good agreement in linear dependency (*R*^2^ ≥ 0.990). Furthermore, the AR_*r*_ distribution increases with the filling volume. Hence, the particle elongation does not seem to correlate with the AR_*r*_ distribution. It is apparent that the *K*_S,*r*_ distribution increases within the intervals that coincide with the region of particle distribution characterised by the highest frequency. This phenomenon can be attributed to the larger number of measurable particles within this range. Conversely, at the boundaries of the particle size distribution, there are considerably fewer particles available for measurement, resulting in a diminished distribution of *K*_S,*r*_ and AR_*r*_ values. Considering the mean widths derived from the TEM analysis, the average *K*_S,*r*_ values calculated with the linear fits are in good agreement with the mean values obtained from the histograms of *K*_S,*r*_. *K*_S,*r*_ is directly proportional to the AR and the size trend obtained by the WH plot is inversely proportional. As the mean size differences between CF7 to CF9 are not significant, the size effect was considered as a non-justifiable fact for the trend of *K*_S_.

In summary, the presentation using size intervals allows for a more accurate visualisation of the *K*_S_ values in relation to the size distribution. Such features, like possible secondary maxima in *K*_S_, would not be as clearly evident using the approach based on eqn (10) (Fig. 5e), but rather through the interval-based representation. However, this has little influence on the calculation of the mean. When considering the standard deviation, it becomes evident that the particle size distribution has a significant effect on *K*_S_ and therefore cannot be neglected within a particle suspension. This is especially important for precipitation reactions or syntheses in aqueous media, which typically exhibit broader size distributions of 10–30%.

#### 3.6.3 Contributions of the strain and the surface anisotropy constants

It is acknowledged that ignoring strain and surface effects raises concerns. Therefore, a brief discussion of their contributions will follow. Strain effects in the particles should be considered due to the ions' distribution above the A- and B-sites of the crystal. Bonvin *et al.* observed increased ordered structures combined with higher crystallinity.^88^ Since the most anisotropic sample, CF9, exhibits the lowest degree of crystallinity of 85%, the influence of the ordered ion distribution should be negligible compared to the other samples. Furthermore, the reaction conditions can influence the strain *ε*, as demonstrated in our previous work, where additional hydrothermal steps under higher pressure of up to 10 bar resulted in increased intrinsic strains.^34^ In this work, no significant increase in the strain obtained *via* WH plot could be detected in all samples (Fig. S4†) compared to cobalt ferrite synthesised hydrothermally, where *ε* ranges from 1.6 × 10^−3^ to 3.0 × 10^−3^.^47,67,88^ However, strain-induced stress in the crystals leads to magnetoelastic anisotropy *K*_*ε*_, which can be calculated by eqn (13)13
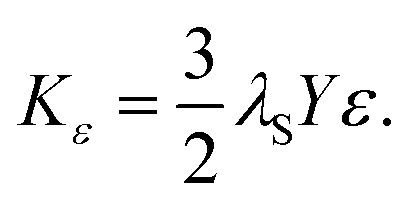
with *λ*_S_ describing the magnetoelastic constant for CF with −7.2 × 10^−5^ composed of the magnetoelastic constants along different crystal directions.^47,86^*Y* denotes the Young's modulus (1.4 × 10^11^ N m^−2^). Eqn (13) assumes that the strain is evenly distributed across the magnetic domains. It is evident that the *K*_*ε*_ values exhibit a similar progression to the strain (Fig. S4†), as a linear relationship exists. Due to the calculation using CF constants from the literature,^47,89^ we obtain relatively high *K*_*ε*_ values in magnitude. These could be overestimated because of the lower cobalt content in the crystal compared to CF as well as to the unidirectional strain assumption. The deviations in determining the strains *via* the WH plot result in relatively high errors in the calculation of the *K*_*ε*_ values, especially for CF7 and CF8. With increasing crystallinity, the error thus increases. This could be related to the assumption that the stress is distributed throughout the entire crystal, which is unlikely at higher degrees of crystallinity. In summary, this value should be interpreted with caution, as strain-induced anisotropy effects are more likely to occur in epitaxial layers or platelets.

It is essential to consider that amorphous regions are evident at the edges of the particles (Fig. 3 and S15†), which may be associated with dead layers at the surface. Assuming a layer thickness of two atoms, or 0.3 nm, this affiliation would correlate with a change in the surface anisotropy *K*_O_.^22,23^ Furthermore, the different sizes associated with the size distribution can be attributed to a change in *K*_O_ previously reported by other workers.^90^ The effective magnetic anisotropy *K* increases with decreasing sizes of the NPs due to a higher contribution of *K*_O_ to *K*.^90^ However, we do not identify significant contributions from surface anisotropy *K*_O_, which was calculated using the magnetic core volume (see Section S10† and Table 2).^91,92^ The magnetic domain size, *d*_mag_, can vary significantly from the particle diameter. This discrepancy is most likely due to a magnetically dead layer present on the surface of the nanoparticles. An increase in the nanoparticle diameter may amplify the effect of surface-spin misalignment, leading to a greater non-magnetic volume and, consequently, a stronger influence on the surface anisotropy constant. As the obtained values for *K*_O_ are seven to eight magnitudes lower than *K*_C_ and *K*_S_, the contribution can be neglected.

#### 3.6.4 Relationship between the determined *K* and heating efficiency

The increase in magnetic anisotropy presumably enhances the hyperthermal effect of nanoparticle suspensions, demonstrating potential applications in biotechnology and medicine. In most studies on magnetic anisotropy, the magneto-crystalline contribution *K*_C_ is initially neglected, as it is anticipated that shape anisotropy will exert a dominant influence or that *K*_S_ will be on a comparable order of magnitude. In this work, we demonstrated that *K*_C_ attains significantly higher values than *K*_S_. However, Faílde *et al.* demonstrated in their simulations, that the highest heating efficiency can be achieved with magnetite particles (*d* = 25 nm) characterised by a slight deviation from a spherical shape, with an AR of 1.12. When examining the mean *K*_S,*r*_ of the samples investigated in this work, CF9 is expected to exhibit the highest heating capacity. Consequently, the specific absorption rate (SAR) values, which are calculated to evaluate the heating capacity, should increase with the magnetic anisotropy of this sample. Further studies should be conducted to elucidate the actual heating behaviour of the more elongated particles presented in this work. The enhancement of SAR by shape-anisotropic particles like magnetite nanocubes or nanorods of similar size and material was already demonstrated in various works.^93–95^ Regardless, some studies indicate that reduced SAR values of spherical CF correspond to increased magneto-crystalline anisotropy compared to spherical magnetite of similar sizes. An enhancement of the SAR values has been realised through the cobalt doping of magnetite to obtain Co_*x*_Fe_3−*x*_O_4_ in the concentration range of *x* < 0.5.^34,96^ This increase can be ascribed to the anisotropic distribution of Co(ii) ions across the tetrahedral and octahedral sites of the inverse spinel lattice, which preferentially accommodates the smaller Co(ii) ions at the octahedral site relative to the substituted Fe(ii) ions, changing the magneto-crystalline anisotropy values. As a result, SAR values should be established not only in relation to factors such as particle phases, sizes, shapes, saturation magnetisations, and dopant concentrations but should also incorporate magnetic anisotropy as a material-dependent parameter. This incorporates both magneto-crystalline anisotropy, which is influenced by, *e.g.*, the material composition and dopant concentration, and shape anisotropy, which is affected by the geometric shape and aspect ratios of the particles. However, when comparing *K* of different shapes of magnetic NPs with the same sizes in an interaction-free environment by adapting well-isolating silica shells, the trend observed is *K*(octahedron) > *K*(sphere) > *K*(cube).^97^ Therefore, the particles studied in this work are expected to exhibit relatively higher *K* values. Simulations by Mamiya *et al.* regarding the contributions of shape, surface, and magneto-crystalline anisotropy for this octahedral particle shape have shown that the purely numerical contribution of shape is the highest and relatively size-independent in the range of 8 nm to 16 nm.^97^ The surface contribution increases with larger particle diameters but remains small in absolute terms, while the magneto-crystalline contribution remains constant across the size range. The distribution of *K*_C_ and *K*_S_, as well as the observed trends with AR, size, and their corresponding distributions, have also been demonstrated for the particles studied in this work.

## 4 Conclusions

This study systematically investigates the synthesis of CFNPs through a coprecipitation reaction combined with a hydrothermal process, utilising akaganeite nanorods as precursors. By carefully adjusting reaction parameters such as temperature, precursor-to-metal salt ratio, and sodium hydroxide concentration, various morphologies from truncated octahedral shapes were successfully obtained. An increase in temperature and a lower precursor-to-metal salt ratio significantly enhanced the shape anisotropy to mean values of up to 1.26 and allowed for a more focused size distribution. The comparison of crystallite sizes from the WH and the HW plot further confirms the consistency and reliability of these findings, where the HW fitting allows *R*^2^ values of up to 0.9998. Additionally, the estimated crystallinity of the synthesised particles indicates that the reaction conditions can be optimised to achieve high degrees of crystalline structures of up to 96% by setting a low precursor-to-metal salt ratio or a high molarity of the base.

Secondly, this work focuses on enhancing the shape anisotropy of truncated octahedral NPs by changing the filling volume of the reactor. Specifically, higher filling volumes correlate with an increase in the proportion of elongated octahedral 3D shapes and elongated honeycomb 2D projections. The HR-TEM and SAED analysis confirmed the nanoparticles' inverse spinel structure and the elongation of the octahedra along the {222} facet.

Furthermore, shape anisotropy constants were determined by assuming a prolate spheroid model, which allowed for an accurate assessment of both magneto-crystalline and shape anisotropy contributions. The results revealed that the anisotropy constant *K*_S_ varies significantly with increasing AR. As expected, shape anisotropy becomes dominant in more elongated particles with a notable increase in *K*_S_ observed as AR exceeds 1.3. The distributions of AR and *K*_S_ also increase with the size distribution. A more precise calculation of the *K*_S_ values through a combination of *M*(*H*) and ACS measurements would be associated with optimising the stabilisation of the particle suspension. This could be achieved through additional stabilisation steps, which would not rely solely on electrostatic stabilisation *via* citrate but could also involve spatial stabilisation using, *e.g.*, inorganic ligands, polymers, and silica shells.

As SAR is inversely proportional to the polydispersity of a sample within the examined single-domain diameter range, it is essential to explore synthetic approaches that result in monodisperse samples with high AR values. Understanding these interdependencies, future studies should aim to enhance the contribution of coffin-like particles with high anisotropy, as these shapes have the potential to significantly improve the performance of magnetic hyperthermia. Extending the experiments to flow reactors could be particularly beneficial, since flow profiles in such systems may positively influence the anisotropy of the particles. Additionally, an experimental investigation of the dependency of the anisotropy of coffin-like particles on hyperthermia efficiency is crucial. This requires more precise magnetic separation of the octahedral and coffin-like morphologies as well as their improved fluidic stabilisation in future studies. Addressing these current limitations and exploring these future perspectives will be essential to enhance the applicability of CFNPs in various biotechnological applications that require optimised magnetic and hyperthermia properties.

## Author contributions

Maria Weißpflog: data curation, analysis, conceptualising, methodology, investigation, roles/writing – original draft, writing – review & editing. Dietmar Eberbeck: analysis, writing – review & editing. Birgit Hankiewicz: supervision, project administration, roles/writing – original draft, writing – review & editing.

## Conflicts of interest

There are no conflicts of interest.

## Supplementary Material

RA-015-D5RA02233A-s001

## Data Availability

The data supporting the results of this study can be found in the article and its ESI.† Should raw data be necessary, it can be made available by the corresponding author upon reasonable request.
