# Supplementary material for: Shape-directed modification of truncated octahedral to coffin-like cobalt-doped ferrite particles by changing the hydrothermal reaction conditions
Source: RSC Adv. 2025 Jul 7;15(28):23007–24. doi: 10.1039/d5ra02233a (PMC12231119; doi:10.1039/d5ra02233a)
Supplement: RA-015-D5RA02233A-s001 [file RA-015-D5RA02233A-s001.pdf]

Supplementary Information (SI) for RSC Advances.
This journal is © The Royal Society of Chemistry 2025

Supplementary Information

Shape-Directed Modification of Truncated Octahedral to Coffin-like Cobalt-doped Ferrite Particles by Changing the Hydrothermal Reaction Conditions

Maria Weißpflog[a], Dietmar Eberbeck[b] and Birgit Hankiewicz[a]

[a] *Institute of Physical Chemistry, University of Hamburg, Grindelallee 117, 20146 Hamburg, Germany*
[b] *Physikalisch-Technische Bundesanstalt (PTB), Abbestraße 2-12, 10587 Berlin, Germany.*

Corresponding contact details: maria.weisspflog@uni-hamburg.de; birgit.hankiewicz@uni-hamburg.de; Tel.: +49 40 42838-8347

Content

S1. Specific conditions for the preparation and stabilisation of cobalt-doped nanoparticles
S2. Analysis of the crystal phases, crystallite sizes, and strains of samples CF1 to CF9
S3. Mechanism
S4. Analysis of the size and aspect ratios of samples CF1 to CF6 using TEM measurements
S5. Analysis of the size and aspect ratios of samples CF7 to CF9 using TEM measurements
S6. Analysis of the shape of samples CF7 to CF9 using angle-dependent SEM measurements
S7. Determination of the crystal planes and *d*-spacings of samples CF7 to CF9 using HR-TEM and SAED measurements
S8. Alternating Current Susceptibility (ACS) measurements for samples CF7 to CF9
S9. Distribution of the shape anisotropy constants by implementing size intervals of samples CF7 to CF9
S10. Determination of the surface anisotropy constants
S11. Additional references

S1. Specific conditions for the preparation and stabilisation of cobalt-doped nanoparticles

In this section, the calculated molar amounts of the metal salts in relation to the molar ratio are summarised in Tab. S1†. The molar ratio of 2 was used for the samples CF1 to CF6, excluding CF5. CF5 was synthesised with a lower molar ratio of 1. The synthesis of CF7 through CF9 was conducted with a precursor-to-metal salt ratio of 0.5. The reactor parameters for the subsequent hydrothermal step are summarised in Tab. S2†. The reactor BR-100 with Teflon inlet from Berghof Instruments GmbH used for particle synthesis, as well as the heating block, are shown in Fig. S1†.

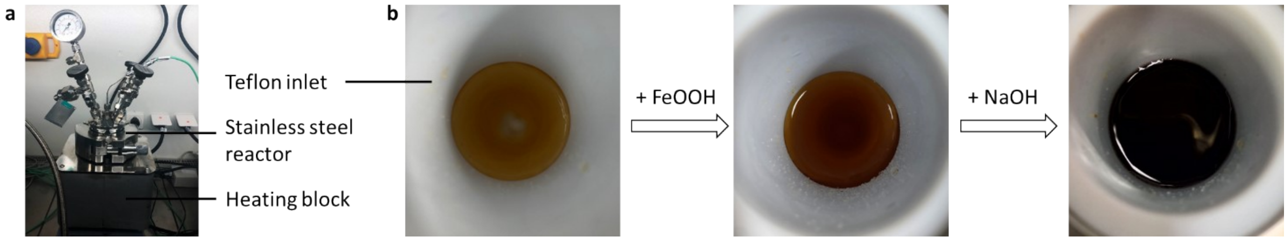

Fig. S1. (**a**) Picture of the hydrothermal reactor BR-100 with Teflon inlet and heating block. (**b**) The yellow metal salt solution changes its color to orange and black by adding the akageneite precursor solution and NaOH solution, respectively.

Tab. S1. The molar ratio *r* of the precursor to the metal salts and the calculated molar amounts *n* and masses *m* of the cobalt, iron(III) and iron(II) chloride salts.

ratio	$n_{precursor}$	$m_{precursor}$	$n_{metal\ salts}$	$n(CoCl_2 \cdot 6H_2O)$	$n(FeCl_3 \cdot 6H_2O)$	$n(FeCl_2 \cdot 4H_2O)$	$m(CoCl_2 \cdot 6H_2O)$	$m(FeCl_3 \cdot 6H_2O)$	$m(FeCl_2 \cdot 4H_2O)$
	**[mmol]**	**[mg]**	**[mmol]**	**[mmol]**	**[mmol]**	**[mmol]**	**[mg]**	**[mg]**	**[mg]**
2	3.376	300	1.688	0.283	0.837	0.568	67.3	226.3	112.9
1	3.376	300	3.376	0.566	1.675	1.135	134.6	452.7	225.8
0.5	3.376	300	6.753	1.132	3.349	2.271	269.3	905.3	451.5

Tab. S2. The reaction parameters of the hydrothermal step. The parameters that differ from CF1 are highlighted in bold.

**No.**	**Molar ratio**	**Solution volume**	**Reactor filling**	**Time**	**Molarity of the base**	**Max. Temperature**	**Pressure**
	**[ ]**	**[%]**	**[%]**	**[hrs]**	**[mol/L]**	**[°C]**	**[bar]**
CF1	2	100	50	24	3	160	1
CF2	2	100	50	**4**	3	160	1
CF3	2	100	50	24	3	**190**	1
CF4	**1**	100	50	24	3	160	1
CF5	2	100	50	24	**5**	160	1
CF6	2	100	50	24	3	160	**10**
CF7	**0.5**	**25**	**17**	24	3	**190**	1
CF8	**0.5**	**50**	**25**	24	3	**190**	1
CF9	**0.5**	100	50	24	3	**190**	1

The subsequent stabilisation process was conducted overnight by dispersing the neutral nanoparticles in 45 mL of 0.25 M tetramethylammonium hydroxide (VWR LLC., Radnor, PA, USA). The excess solvent was evaporated to achieve a mass fraction of 1.0 wt.%. The concentrated dispersion was then mixed with 100 mM citric acid (Grüssing, Filsum, Germany) to reach a target weight percentage of 0.25 wt.% and stirred for two hours. The particles were isolated *via* magnetic decantation and resuspended in an equal volume

of 20 mM trisodium citrate (Fluka Inc., Buchs, Switzerland) overnight. The stabilised solutions were dialysed against milli-Q water for three days, and the water bath was changed twice a day. The particle dispersions were concentrated to 1.0 wt.% particle mass fraction by evaporating at 40 mbar and 40 °C. The samples sedimented quickly, from 10 minutes to 24 hours, despite the stabilisation process. Before the VSM measurements, all samples were processed ultrasonically for 1 minute at 90% power to increase the stability.

S2. Analysis of the crystal phases, crystallite sizes, and strains of samples CF1 to CF9

The diffraction patterns of the samples CF1 to CF6 (Fig. S2†) and CF7 to CF9 (Fig. S3†) are depicted in reference to cobalt ferrite (JCPDS PDF no. 00-003-0864).

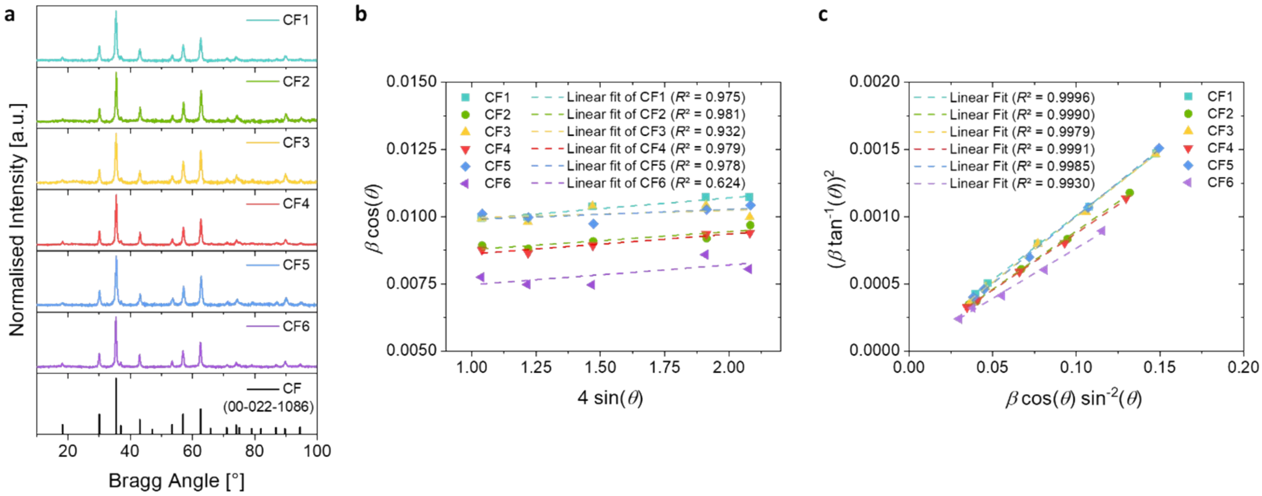

Fig. S2. (a) The diffractograms, **(b)** the WH plots, and **(c)** the HW plots of the samples CF1 to CF6 are depicted. The JCPDS PDF no. 00-003-0864 of cobalt ferrite is referenced as well.

The only deviation resulting from an additional phase is observed in CF9, where supplementary reflections are evident at 10.9°,18.8°, 28.6°, and 30.0°. These reflections can be assigned to the monoclinic phase of trisodium citrate dihydrate, which was utilised in the stabilisation step of the nanoparticles.[S1] It appears that the subsequent dialysis process was not fully completed, leading to the emergence of these additional citrate reflections.

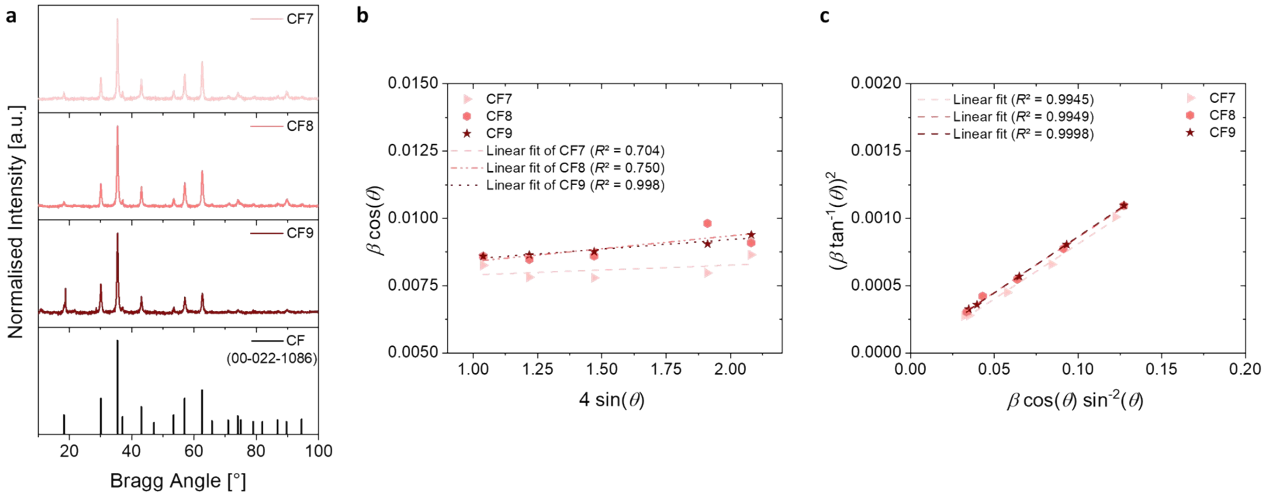

Fig. S3. (a) The diffractograms, **(b)** the WH plots, and **(c)** the HW plots of the samples CF7 to CF9 are depicted. The JCPDS PDF no. 00-003-0864 of cobalt ferrite is referenced as well.

The corresponding WH plots are figured as well, where the calculation of the crystallite size d_{WH} was obtained from the ordinate intercepts. The slope of the linear fit provides insights into the intrinsic strain (Fig. S4†). Using an alternative formula by the Halder-Wagner method, the crystallite sizes d_{HW} were calculated from the slope and the strain from the intercepts. A clear trend cannot be discerned that would allow conclusions about the dependence of strains on the reaction parameters. Overall, these values are very small and positive for both methods, indicating a stretching effect on the crystal lattice. This effect is observed in comparison to pure magnetite when cobalt(II) ions are incorporated, which have a smaller radius than iron(II) ions. As the cobalt content in the crystal lattice increases, so does the strain. In addition to the cobalt concentration (*i.e.*, the concentration of metal salts), the reaction temperature also influences the crystal structure. CF3 (190°C) exhibits a lower strain compared to the samples synthesised at 160°C. This may be related to the reduction of crystal defects associated with oxygen vacancies.

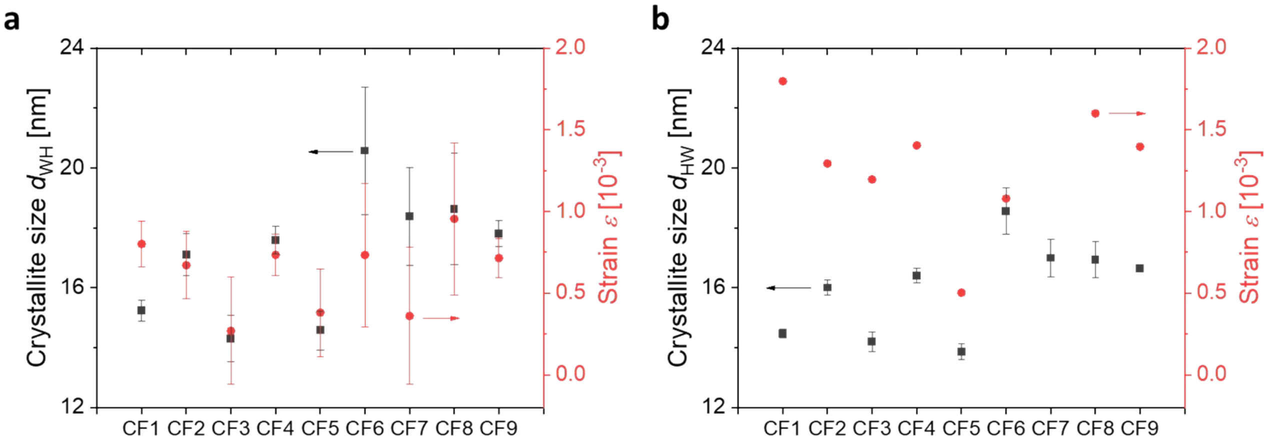

Fig. S4. The crystallite size and intrinsic strain values of the nanoparticle samples are obtained from the (a) WH and (b) HW plots, respectively. The error bars are depicted as well.

The crystallinity index (*CI*) for each sample was calculated using Eq. S1:

$$CI = A_c/A \cdot 100\%. \quad \text{(S1)}$$

Therefore, the total area A and the sum of the areas of the crystalline peaks A_c were integrated for each diffractogram (Fig. S5†).

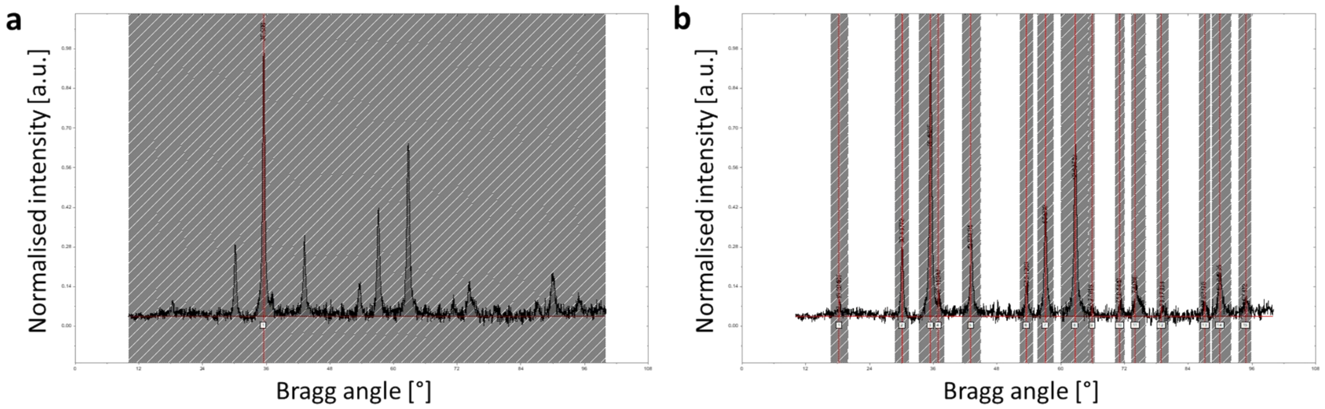

Fig. S5. (**a**) The total area of all peaks and (**b**) the sum of the area of the crystalline peaks of the samples CF7 to CF9 are depicted.

S3. Mechanism

TEM and diffraction images of the initial phase of the coprecipitation reaction are shown in Fig. S6† as a function of time after the addition of the NaOH solution. The stirring time at room temperature is shown here up to 300 s, which represents the approximate maximum time required to close the reactor and start the heating program. Akaganeite particles are still present after the addition of NaOH solution. Furthermore, small spheres are produced, indicating the presence of magnetite nuclei from the Massart reaction. The presence of akaganeite rods after 24 hours of stirring at room temperature is demonstrated in our previous work.[35]

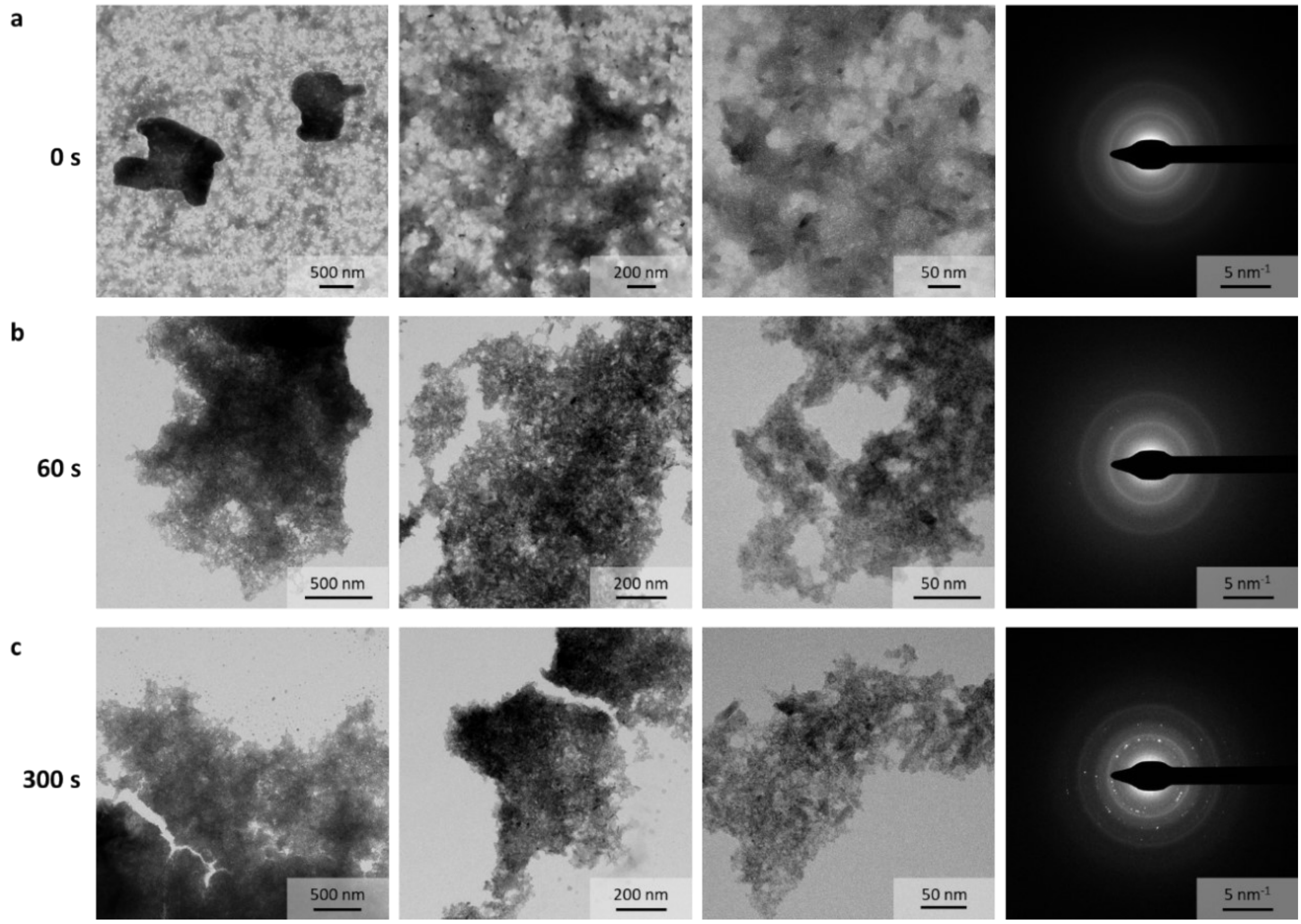

Fig. S6. TEM and diffraction images of the initial phase of the coprecipitation reaction are shown as a function of time for (**a**) 0 s (**b**) 60 s, and (**c**) 300 s after the addition of the NaOH solution.

If the reaction solution is heated to 90°C after the initial time of 300 s and then opened directly, changes can be observed in the TEM images (Fig. S7†). Cubic (2D projection) particles appear, which may indicate octahedral shapes. If this reaction solution is now allowed to stir at room temperature (total reaction time 24 h), particles in octahedral and spherical form are recognizable simultaneously. Higher temperatures than 90°C (for cobalt ferrite formation) are therefore required to increase crystallinity and single-phase formation.

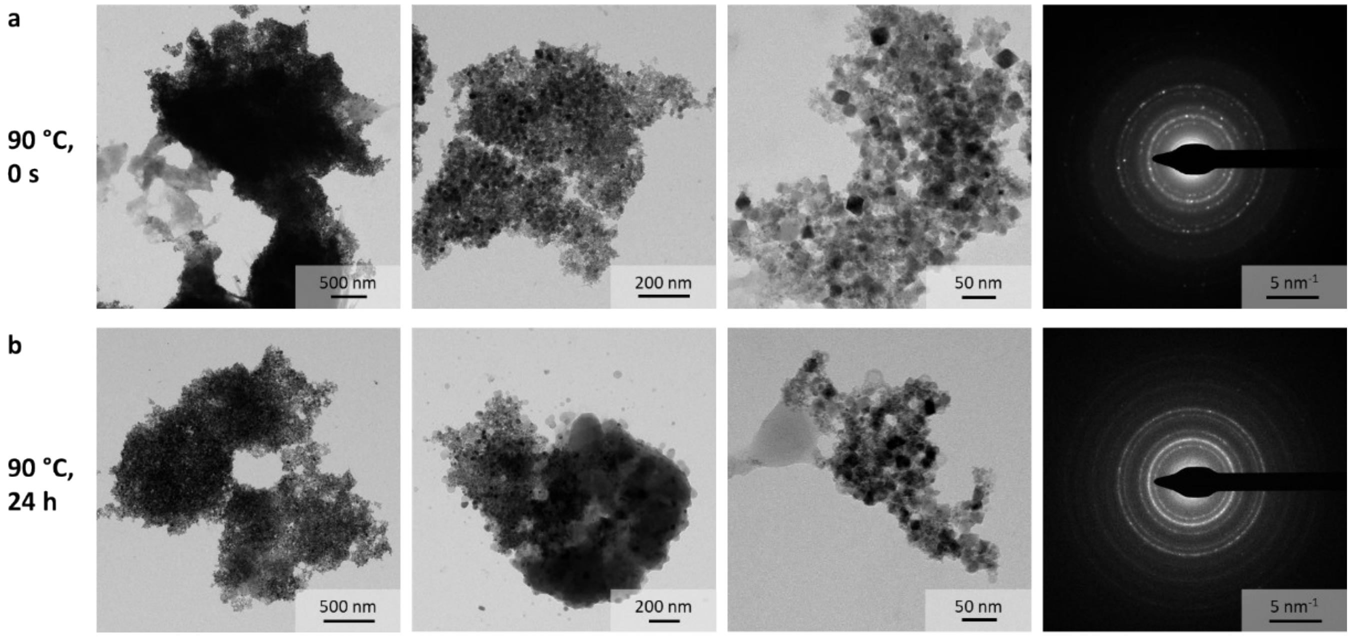

Fig. S7. When the reaction solution is heated to 90°C after the initial time of 300 s and then opened, samples are examined (**a**) directly and (**b**) after 24 hours of stirring at room temperature.

S4. Analysis of the size and aspect ratios of samples CF1 to CF6 using TEM measurements

In this section, the histograms of the measured lengths (Fig. S8a†) and the widths (Fig. S8b†) are depicted. The calculated diameters are shown in the manuscript in Fig. 2b.

The fitting of the histograms was performed on a logarithmic scale. A log-normal distribution is a probability distribution where the logarithmic values of a variable are normally distributed. This means that when the particle sizes (*e.g.*, diameters) are displayed logarithmically, they form a symmetric bell-shaped curve. The original sizes then follow a right-skewed distribution, with larger particles being less frequent but still possible. In contrast, the normal distribution is symmetric around the mean, with most values near it and decreasing probability for larger deviations. In nanoparticle synthesis *via* coprecipitation (followed by hydrothermal processes), the log-normal distribution is particularly well-suited to describe the variability in particle sizes. Since growth processes are influenced by multiplicative factors, the resulting particle sizes often follow a log-normal distribution. This distribution is characteristic because it captures both the frequently occurring average particle sizes and the occasionally larger particles that arise from different growth dynamics, such as Ostwald ripening or its extended version. Therefore, the log-normal distribution is excellent for modeling the often asymmetric size distribution observed in nanoparticle populations.

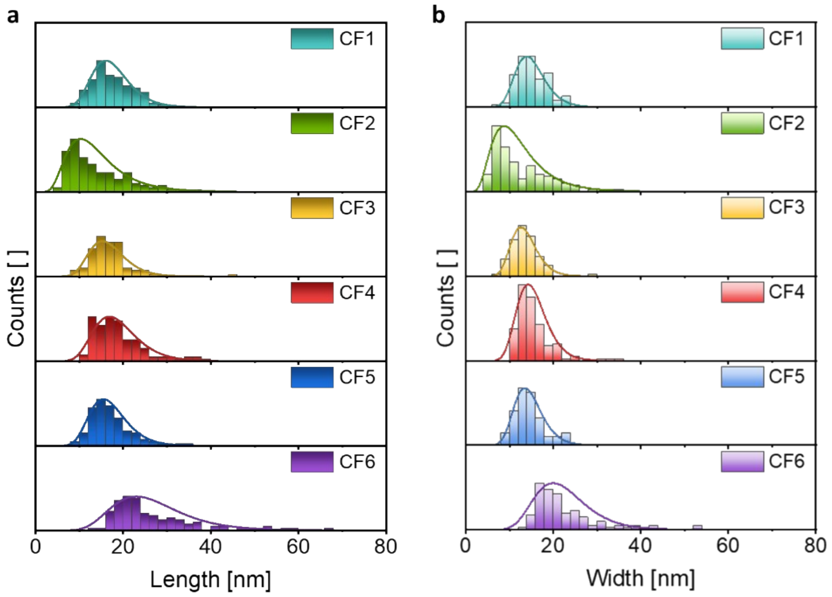

Fig. S8. (**a**) The lengths and (**b**) the widths of the samples CF1 to CF6 are depicted as histograms with the lognormal distribution curves.

The determination of the *AR* was made using Eq. 4 (Fig. 2c). Additionally, the distribution of the diameters is shown in dependence on the *AR*, which was distributed over the values above and below 1.2 where we expect significantly higher shape anisotropy (Fig. S9†). Magnetite, for example, shows high shape anisotropy constant values above *AR* values of 1.05 to 1.12 (see references Clarke *et al.*[22] and Faílde *et al.*[28]).

The decrease of the molarity of the base to 1 mol/L is comparable to the small reactor experiments using BR-25, which were conducted by our group in previous work (see reference Weißpflog *et al.*[35]).

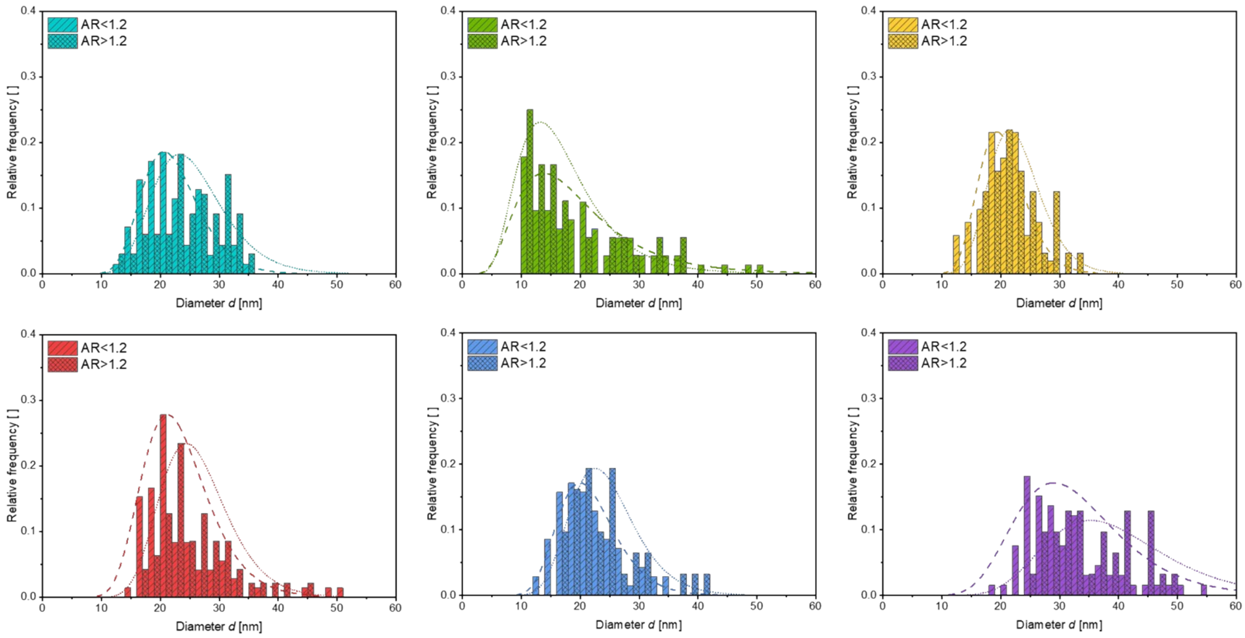

Fig. S9. The diameter distributions of the samples CF1 to CF6 are depicted as histograms with the lognormal distribution curves for two contributions differentiated between an *AR* value below and above 1.2.

The distributions of the diameters in dependence of *AR* are shown in Fig. S10†, where the data point sizes are also depicted in relation to the diameter. In comparison, the particle shape is irregular and the sample shows a bisdispere character (Fig. S11†).

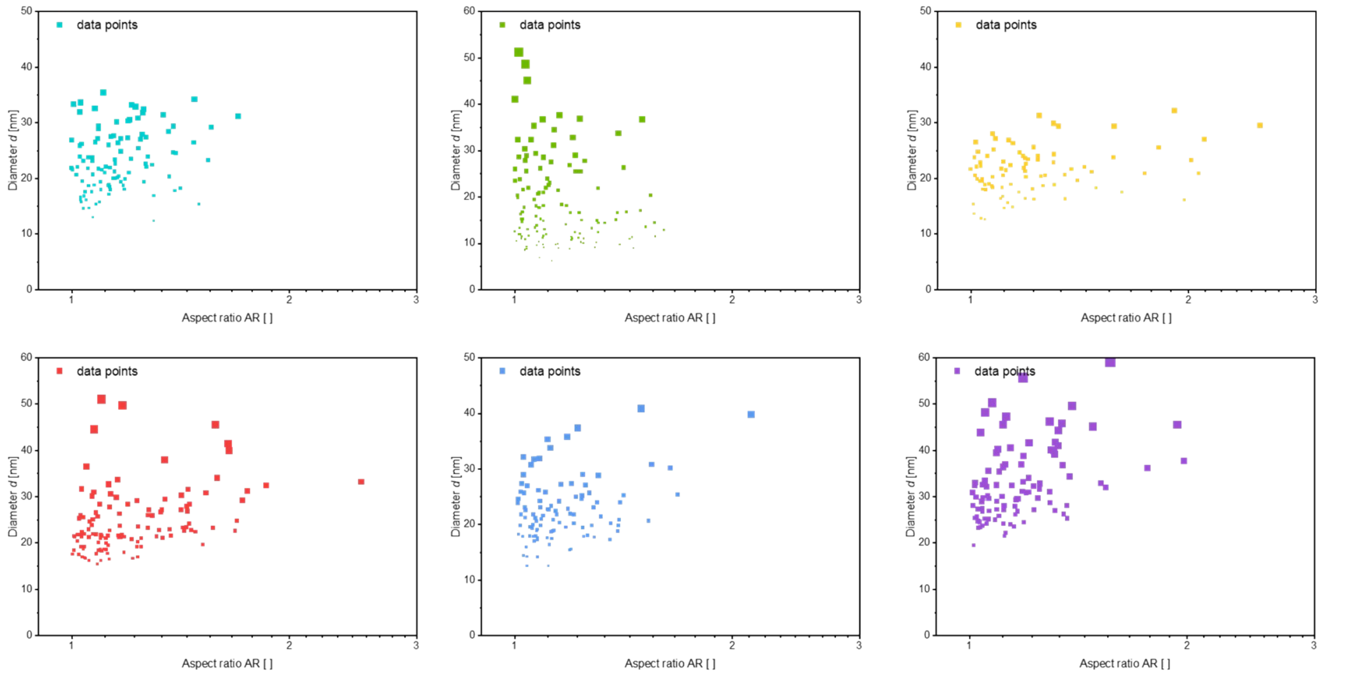

Fig. S10. The diameters of the samples CF1 to CF6 are shown based on the aspect ratio. The sizes of the data points are scaled according to the diameters.

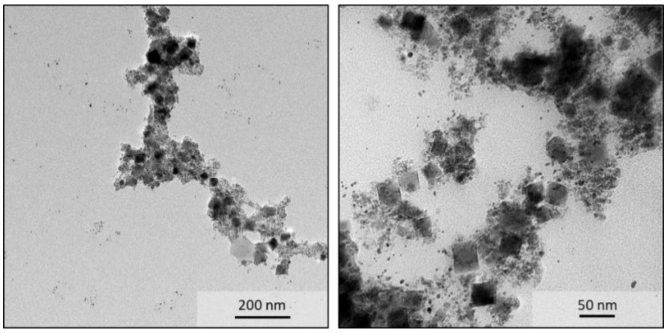

Fig. S11. TEM images of the sample synthesized with 1 mol/L base resulting in a bidisperse nanoparticle solution.

The PDI is calculated using the formula (size/standard deviation)² assuming a Gaussian distribution (Tab. S3†). This normal-distribution assumption is less accurate for the nanoparticle formation mechanism, but gives a good approximation of the size-to-deviation ratio.

Tab. S3. Summary of the PDI of the samples CF1 to CF6.

No.	PDI$_w$	PDI$_d$	PDI$_{WH}$
	[ ]	[ ]	[ ]
CF1	0.06	0.06	<0.01
CF2	0.26	0.24	<0.01
CF3	0.06	0.04	<0.01
CF4	0.05	0.18	<0.01
CF5	0.08	0.06	<0.01
CF6	0.05	0.09	0.01

S5. Analysis of the size and aspect ratios of samples CF7 to CF9 using TEM measurements

The histograms illustrate the measured widths (Fig. S12a†), lengths (Fig. S12b†), and the calculated diameters (Fig. S12c†). The fitting of the histograms was performed on a logarithmic scale as explained in Sec. S4†. The *AR* distribution (Fig. S12d†) was determined using Eq. 3. Furthermore, the diameter distribution is displayed in relation to the *AR*, using a boxplot illustration (Fig. S12e†). The PDI is calculated using the formula (size/standard deviation)^2 (Tab. S4†).

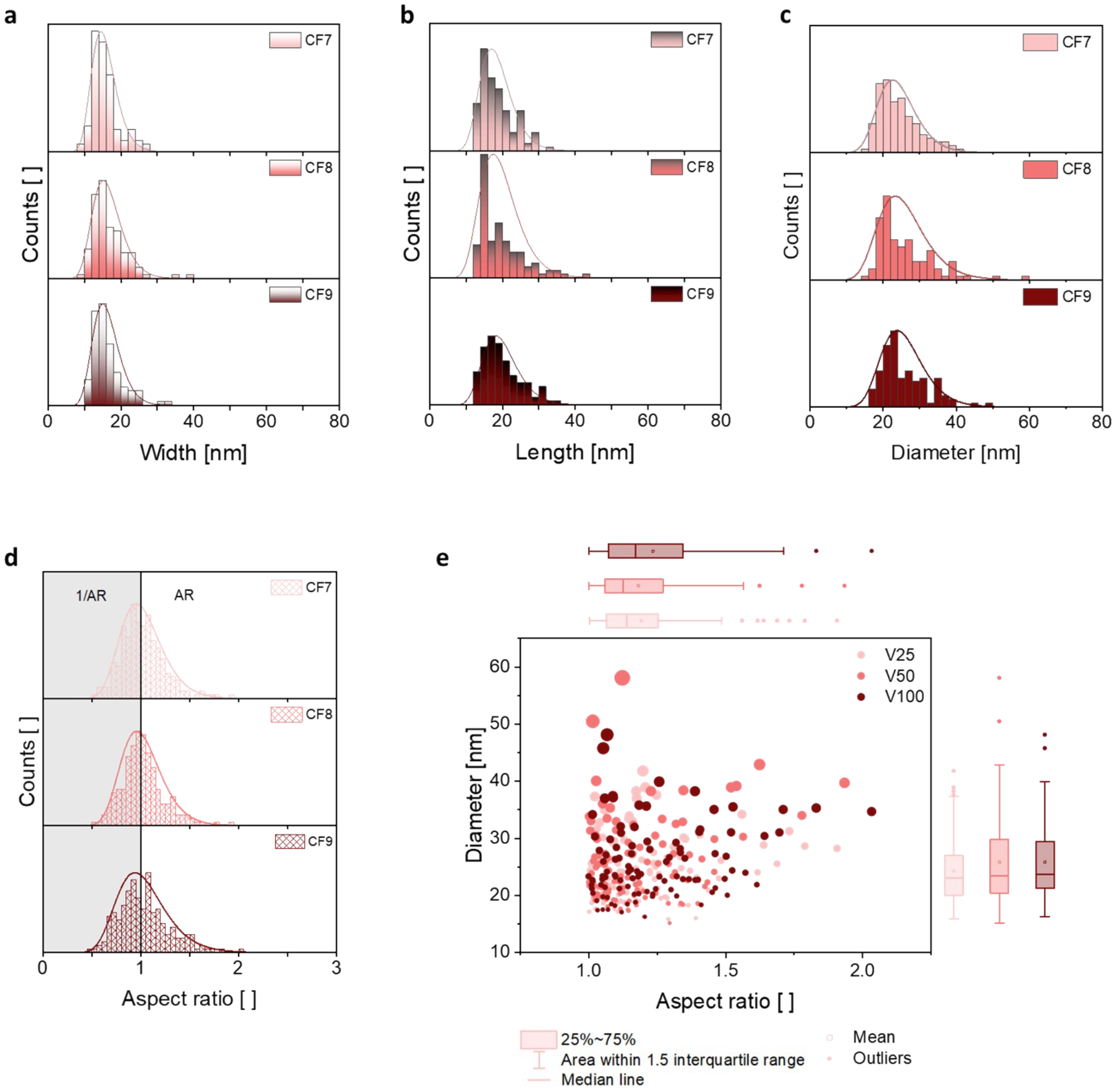

Fig. S12. (a-c) The sizes and (d) the aspect ratios of the samples CF7 to CF9 are depicted as histograms with the lognormal distribution curves. (e) The dependency of the *AR* on the diameters is shown as a Boxplot illustration.

Tab. S4. Summary of the PDI of the samples CF7 to CF9.

No.	PDI_w	PDI_l	PDI_d
	[ ]	[ ]	[ ]
CF7	0.05	0.05	<0.01
CF8	0.06	0.07	0.01
CF9	0.05	0.05	<0.01

S6. Analysis of the shape of samples CF7 to CF9 using angle-dependent SEM measurements

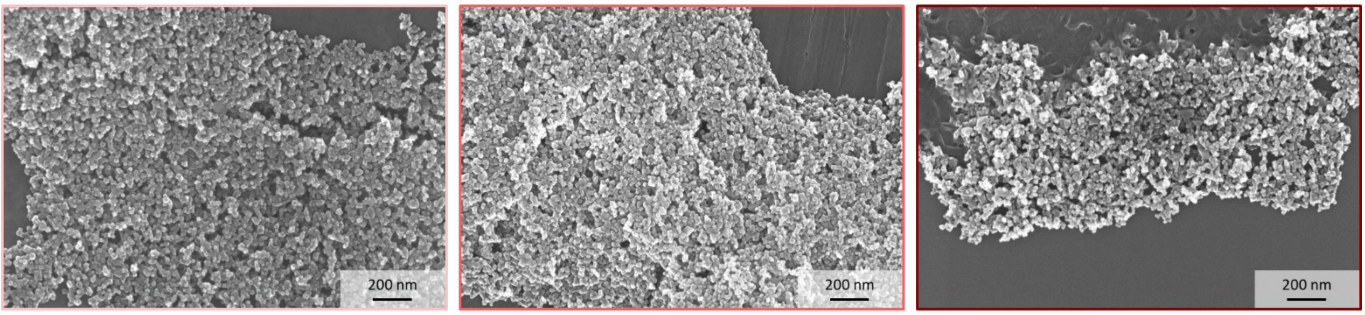

Fig. S13. SEM images with a magnification of 50.000x at a tilt angle of 0° of the samples CF7 to CF9 (from left to right).

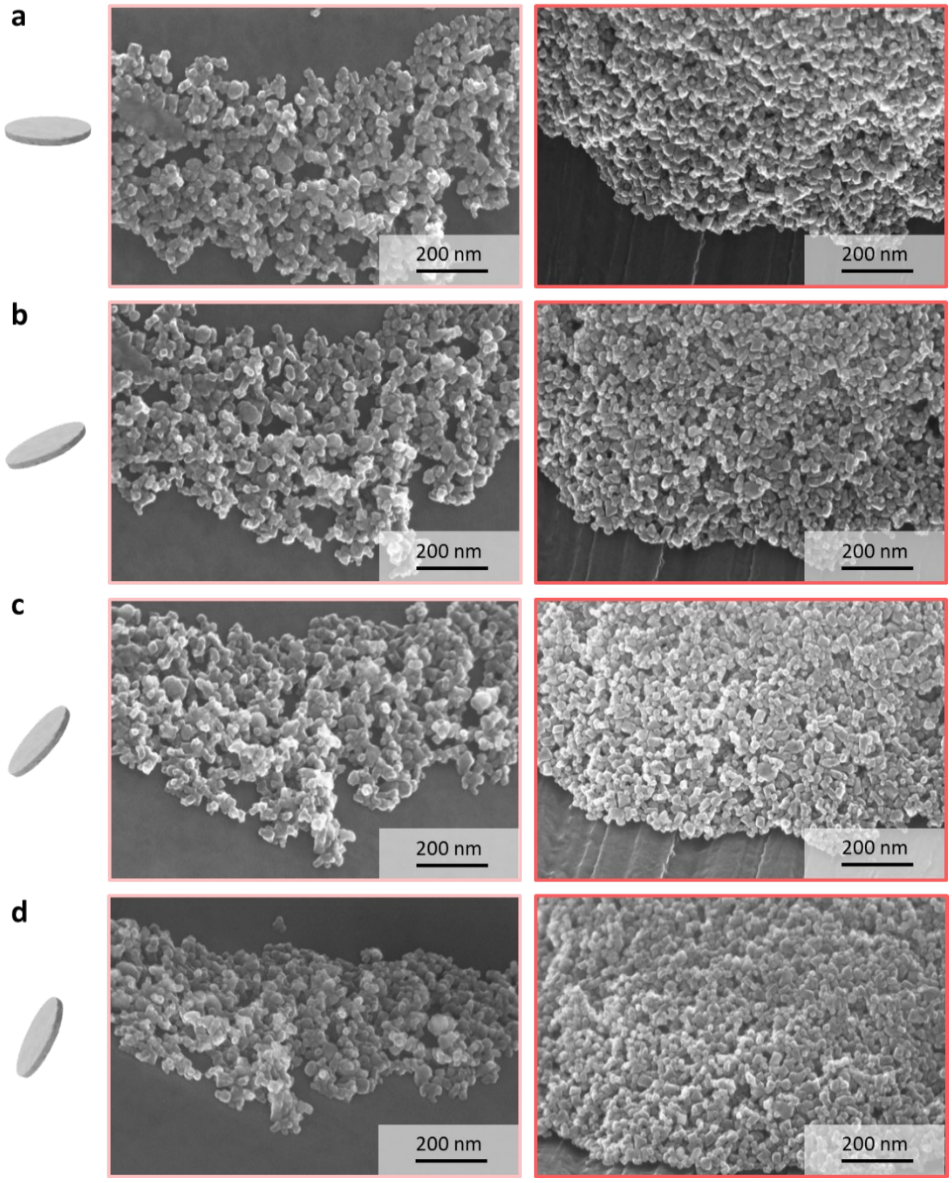

Fig. S14. SEM images with a magnification of 100.000x at a tilt angle of (**a**) 0°, (**b**) 25°, (**c**) 50°, and (**d**) 70° of the samples CF7 and CF8 (from left to right).

S7. Determination of the crystal planes and *d*-spacings of samples CF7 to CF9 using HR-TEM and SAED measurements

In this chapter, the interplanar spacings and lattice parameters from the crystalline structure of individual nanoparticles are provided, which were calculated during the analysis of the HR-TEM images (Fig. S15†).

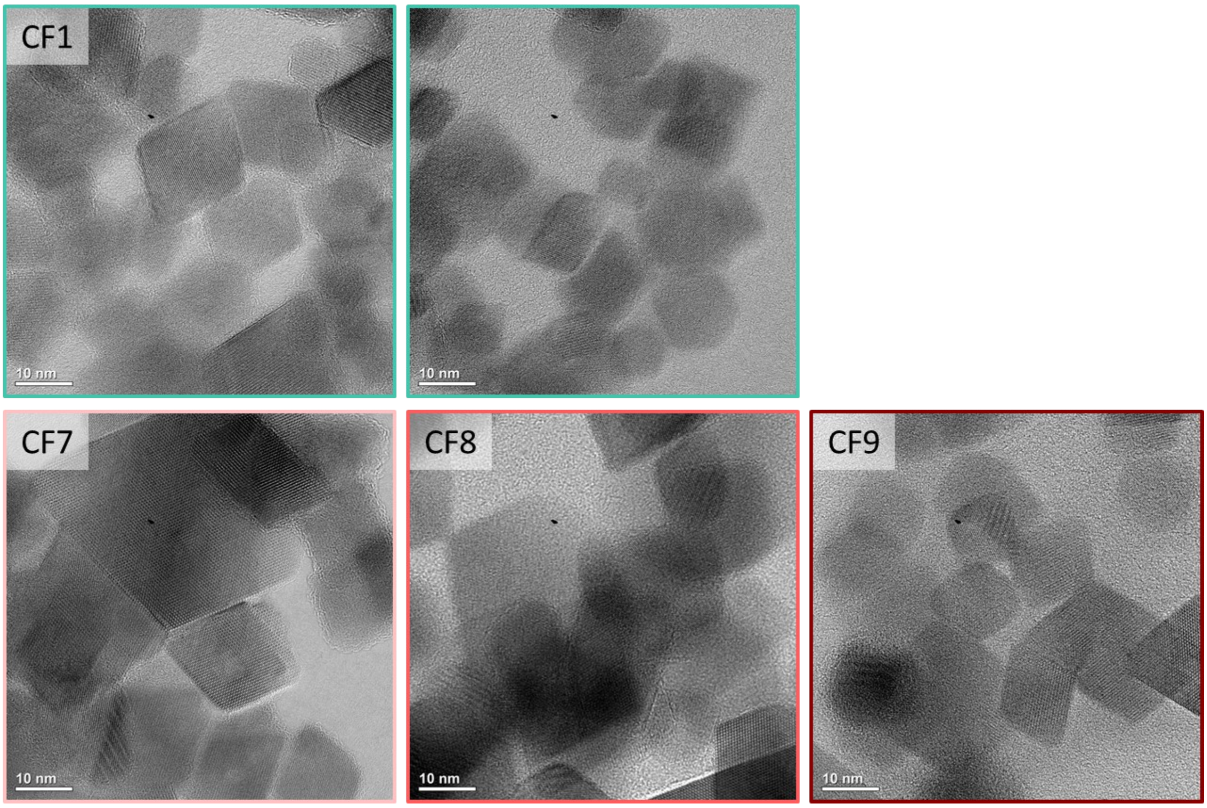

Fig. S15. Additional HR-TEM images. Three additional exemplary HR-TEM images of the samples CF1 and CF7 to CF9 are shown which were used for calculation of the *d*-spacings and lattice parameters.

For this purpose, a Fast Fourier Transformation (FFT) (Fig. S16†) was performed to visualise the diffraction pattern. This also allows for the assessment of the crystallinity of the sample.

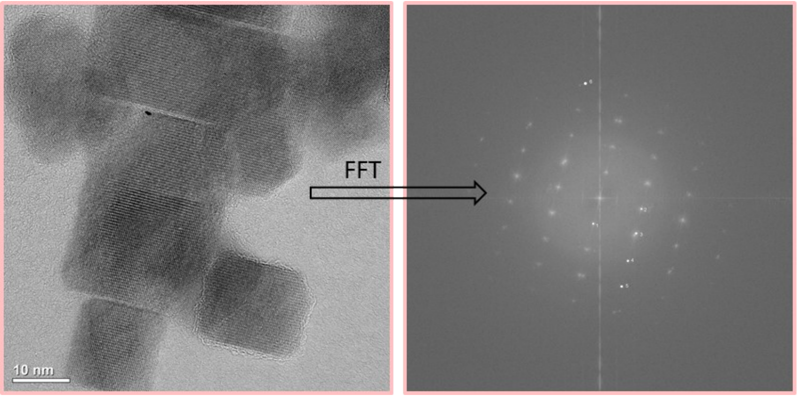

Fig. S16. Fast Fourier Transformation (FFT). An exemplary HR-TEM image of sample CF7 (left) is depicted. The diffraction spots in the pattern (right) are numbered.

Subsequently, an inverse FFT image is generated (Fig. S17†), which is formed by selecting a specific diffraction spot and can be assigned to the particular particles. One advantage of this process is that this inverse FFT image will show the inter-spacings between the atomic layers with higher contrast.

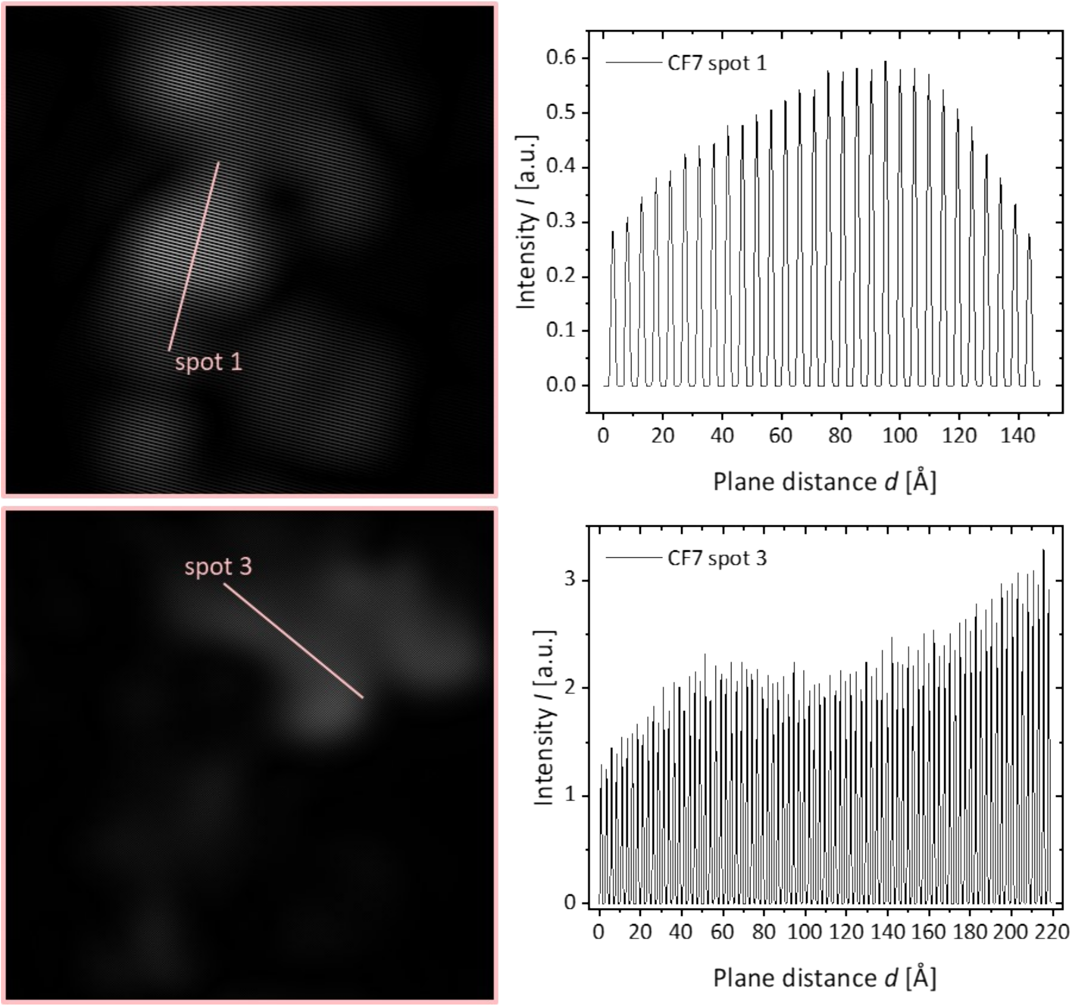

Fig. S17. Inverse Fast Fourier Transformation (IFFT). The IFFT images are depicted for the diffraction spots 1 and 3 (left). The plot profiles of the lines perpendicular to the planes are used for the calculation of the *d*-spacings and the errors (right).

The resulting plot profiles, which are created perpendicular to the atomic layers, enable the calculation of the distances between the maxima, which correspond to the *d*-spacings $d_{hkl,HRT}$ (Tab. S5†).

The diffraction patterns were analysed by measuring the radii of the diffraction rings and calculating the *d*-spacings $d_{hkl,SAED}$ as the reciprocal values, which are summarised in Tab. S6†. The corresponding lattice parameter *a* of the samples was calculated using Eq. S2†, assuming a cubic crystal structure.

$$a = \sqrt{d_{\mathrm{hkl}}^2 \cdot \left(h^2 + k^2 + l^2\right)}. \qquad \text{(S2)}$$

Tab. S5. The calculated *d*-spacings $d_{hkl,HRT}$ lattice parameters a_{HRT} for a cubic crystal structure, and the assigned (hkl) planes of the samples CF1 and CF7 to CF9 obtained by the FFT- and inverse FFT process described above are summarized in comparison to the cobalt ferrite reference from JCPDS PDF no. 00-022-1086.

(hkl)	d_{hkl}(CF)	a(CF)	$d_{hkl,HRT}$(CF1)	a_{HRT}(CF1)	$d_{hkl,HRT}$CF7)	a_{HRT}(CF7)	$d_{hkl,HRT}$(CF8)	a_{HRT}(CF8)	$d_{hkl,HRT}$(CF9)	a_{HRT}(CF9)
	[Å]	[Å]	[Å]	[Å]	[Å]	[Å]	[Å]	[Å]	[Å]	[Å]
111	4.847	8.395	4.82 ± 0.17	8.354	4.85 ± 0.17	8.393	4.85 ± 0.17	8.416	4.85 ± 0.17	8.396
220	2.968	8.395	2.99 ± 0.05	8.479	2.98 ± 0.08	8.438	2.98 ± 0.13	8.436	2.95 ± 0.14	8.436
311	2.531	8.394	2.53 ± 0.16	8.395	2.53 ± 0.16	8.379	2.51 ± 0.17	8.435	2.54 ± 0.16	8.317
222	2.424	8.397	2.42 ± 0.76	8.395	2.42 ± 0.15	8.377	2.40 ± 0.14	8.391	2.42 ± 0.15	8.315
400	2.099	8.396	2.07 ± 0.14	8.292	2.12 ± 0.16	8.483	2.09 ± 0.15	8.356	2.10 ± 0.16	8.402
422	1.713	8.392	1.73 ± 0.13	8.458	1.70 ± 0.11	8.343	1.71 ± 0.11	8.370	1.71 ± 0.12	8.344
511	1.615	8.392	1.64 ± 0.10	8.501	1.61 ± 0.12	8.377	1.60 ± 0.13	8.332	1.61 ± 0.12	8.365
440	1.483	8.389	1.50 ± 0.17	8.468	1.47 ± 0.51	8.307	1.47 ± 0.17	8.320	1.47 ± 0.16	8.313

Tab. S6. The calculated *d*-spacings $d_{hkl,SAED}$ lattice parameters a_{SAED} for a cubic crystal structure, and the assigned (hkl) planes of the samples CF1 and CF7 to CF9 obtained by the diffraction patterns are summarised in comparison to the cobalt ferrite reference from JCPDS PDF no. 00-022-1086.

(hkl)	d_{hkl}(CF)	a(CF)	$d_{hkl,SAED}$(CF1)	a_{SAED}(CF1)	$d_{hkl,SAED}$(CF7)	a_{SAED}(CF7)	$d_{hkl,SAED}$(CF8)	a_{SAED}(CF8)	$d_{hkl,SAED}$(CF9)	a_{SAED}(CF9)
	[Å]	[Å]	[Å]	[Å]	[Å]	[Å]	[Å]	[Å]	[Å]	[Å]
111	4.847	8.395	4.857	8.412	4.857	8.412	4.852	8.404	4.850	8.400
220	2.968	8.395	2.976	8.418	2.967	8.393	2.974	8.413	2.968	8.395
311	2.531	8.394	2.537	8.414	2.530	8.390	2.543	8.433	2.528	8.384
400	2.099	8.396	2.107	8.426	2.108	8.432	2.104	8.416	2.091	8.365
422	1.713	8.392	1.715	8.402	1.713	8.393	1.720	8.425	1.714	8.397
511	1.615	8.392	1.616	8.399	1.614	8.385	1.615	8.393	1.617	8.403
440	1.483	8.389	1.484	8.395	1.482	8.383	1.489	8.422	1.485	8.399
533	1.280	8.392	1.279	8.387	1.278	8.381	1.280	8.394	1.277	8.374

S8. Alternating Current Susceptibility (ACS) measurements for samples CF7 to CF9

The anisotropy constant of non-interacting magnetic NP (MNP) can estimated from the coercivity field H_c measured at low temperatures which is determined in the case of uniaxial anisotropy to $H_c = 0.96\, K/(\mu_0 M_s)$, where thermal agitation is not taken into account within the Stoner-Wohlfarth model.[S2] K may also be estimated from dynamic magnetic measurements, like for example, Magetorelaxometry[S3] or Alternating Current Susceptibility (ACS).[S4] The quasistatic *M(H)* with its long time constant of magnetic excitation has the disadvantage to more substantial support of long-range and long-term magnetic correlations in case of a (residual) Dipole-Dipole-Interaction (DDI), which is not easy to suppress because of its long-range property. Hence, here we harnessed the ACS to estimate the effective anisotropy constant K in terms of a uniaxial anisotropy.

Methods:

For the ACS measurement, the diluted MNP dispersions were filled in glass tubes. The samples were then measured using an AC-susceptometer DynoMag[TM] (Acreo, Sweden). The used frequency range was

f=10 Hz ... 500 kHz. The excitation field was about 350 A/m at $f < 10\ kHz$ and decreases down to 300 A/m towards higher frequencies. The measurements run at ambient temperature. The temperature was measured near the sample cuvette.

The susceptibility of MNP within an alternating magnetic field is determined by its magnetic moment $\mu = M_s V_c$, with the saturation magnetization M_s and the core volume V_c and its mobility which in turn is parameterized by the relaxation time.

The dynamics of the magnetic moments can be characterised by the effective relaxation time

$$\tau_{eff} = \frac{\tau_B \tau_N}{\tau_B + \tau_N} \tag{S3}$$

where

$$\tau_N = \tau_0 exp\left(\frac{KV_d}{k_B T}\right) \tag{S4}$$

represents the dynamics of the Néel relaxation of the moment within the domain. V_d, K and k_B are the volume of the particle domain, the anisotropy constant and the Boltzmann constant, respectively. τ_0 was set to $10^{-10} s$.[S5] Here we assumed single domain MNP, *i.e.* $V_d = V_c$.

$$\tau_B = \frac{3\eta V_h}{k_B T} \tag{S5}$$

is the time constant of the Brownian relaxation of the whole particle with the hydrodynamic volume $V_h = \pi/6\ d_h^3$ which is determined by the core diameter d_c, the thickness of the non-magnetic shell δ_s, and a solvation layer δ_{sol} (in aqueous media).

$$d_h = d_c + 2\delta_s + 2\delta_{sol} \tag{S6}$$

The susceptibility reads

$$\chi(\omega) = \frac{M_s}{H} \frac{1}{\bar{d}_d^3} \int \frac{f(\tilde{d}_c, \sigma_c, d_c) d_d^3(d_c) L(M_s, d_c, T, H)}{1 - i\omega\tau_{eff}(d_h(d_c), K, M_s, d_c)} dd_c \tag{S7}$$

where $d_h(d_c)$ is given by Eq S6.

For the final fit, we used

$$\chi = A(\phi_N \chi_N + (1 - \phi_N)\chi_B) \tag{S8}$$

where A is a normalised amplitude fit parameter, the value of which is one for a quantitatively correct model. χ_N describes the susceptibility of immobilised MNP, the moments which relax by Néel relaxation mechanism (Eq. S4) only. While χ_B represents the susceptibility of the MNP, which may rotate *via* Brownian mechanism (Eq. S5). ϕ_N stands for the fraction of immobilised MNP.

The MNP systems CF7 and CF8 were measured in its dispersion and were also immobilised in a polyacrylamide gel prior to the ACS measurement. It was observed that the MNP within the dispersions precipitate within a time range of about 10 minutes.

While the ACS data of the fluid CF8 sample (dispersion, Fig. S18b) shows a typical Debye behaviour of Brownian MNP superimposed with a continuously declining part. This latter part is typical for moments with a very broad distribution of relaxation times and can be attributed to immobilised MNP, the relaxation time distribution of which is very broad, even if the core size distribution is rather narrow, because of the exponential dependence in Eq. S4. Also, in the case of DDI within disordered systems the distribution of relaxation times may be very broad due to the onset of spin glass-like behaviour.[S6]

The fit of Eq. S8 was performed under the constraint of effective sphere diameters and dispersion parameter (both extracted from the values of Tab. 1) for the median core size $\bar{d}_c$ and σ_c, respectively. The constraint for the saturation magnetisation M_s was taken from Tab. 2. For the distribution f a lognormal function was applied. The results show that the values for K of the different states (dispersion, immobilised of the samples differ by a factor of about 2 (Tab. S7). This is likely caused by dipolar interaction, which in turn may be different for the two states. Furthermore, the fraction of apparent bound particles, *i.e.* Néel particles, ϕ_N, is about 100% in the dispersion of CF7 (Fig. S18a) while within the dispersion of CF8 remains a significant fraction of Brownian MNP of $\phi_B = 1 - \phi_N = 35\%$, despite on the visually almost complete precipitation of the MNP. This is related to the mentioned Debye behaviour of the $\chi(f)$ in Fig. S18c. Nevertheless, the ratio of the corresponding K-values between CF7 and CF8 matches the ratio of the related K_c-values from Tab. 2.

The ACS curves of CF9 show a more complicated structure (Fig. S18c). They may be apparently described by Brownian objects, which comprise 33% single MNP plus 67% clusters with a very broad size distribution, and by 40% Néel particles. However, we have doubts about even the rough correctness of the values of some of these parameters.

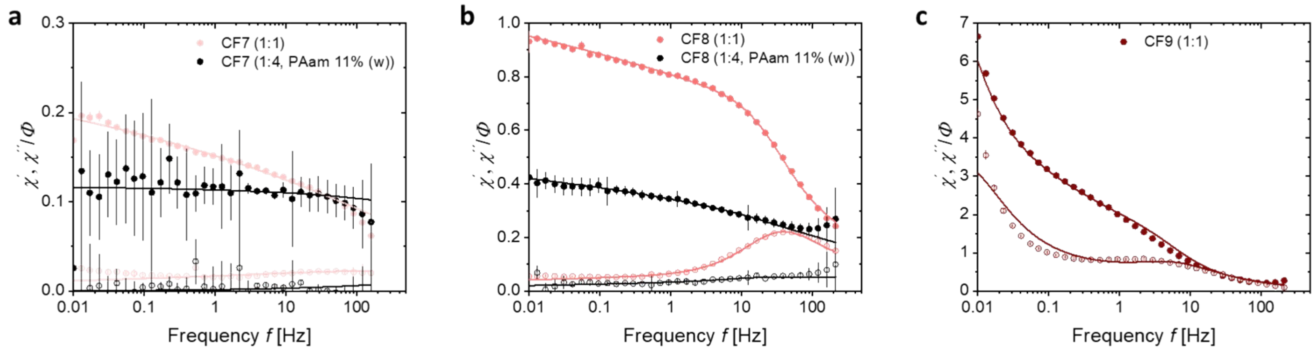

Fig. S18: ACS-data and fit results for (**a**) CF7, (**b**) CF8, and (**c**) CF9 in dispersion and additionally embedded in polyacrylamide (PAam) for CF7 and CF8. The filled data points represent the real part, and the hollow data points the imaginary part. The lines represent the best fit results.

As a striking result appears the smallness of the amplitude parameter A which should be in the range of 1. This result strongly supports the hypothesis of the appearance of aggregates of MNP, where its moments seem to arrange in flux closure-magnetic structure strongly reducing the stray field of these aggregates.

We conclude that the anisotropy constant of MNP cannot be estimated with sufficient accuracy on the basis of an integral measurement method like ACS because of the dipolar interaction between the magnetic moments of MNP organised within aggregates. Additionally, other methods, such as FMR, are not currently usable. That's why we calculate the anisotropy constant in the publication using a LAS model for K_C combined with a calculation of K_S assuming a spheroid.

Tab. S7: Parameters of the best fit for CF7 and CF8 in aqueous dispersion (H_2O) and embedded in polyacrylamide. As indicated, a constraint was admitted for some parameters.

Sample	Matrix	A	d_m	$\bar{d}_m$	σ_m	M_S	K	ϕ_N
			nm	nm		kA/m	kJ/m³	
constraint CF7 →			16.6 ± 0.5		0.22 ± 0.01	76 ± 10		
CF7	H_2O	0.092 ± 0.032	16.5 ± 0.6	17.2 ± 0.8	0.26 ± 0.01	76 ± 13	7 ± 1	100 ± 2
	PAam	0.043 ± 0.013	16.6 ± 0.5	17.0 ± 0.7	0.23 ± 0.01	76 ± 10	3 ± 1	100 ± 114
constraint CF8 →			17.5 ± 0.5		0.23 ± 0.01	142 ± 10		
CF8	H_2O	0.230 ± 0.044	15.0 ± 0.4	15.4 ± 0.5	0.22 ± 0.01	142 ± 12	16 ± 1	65 ± 1
	PAam	0.060 ± 0.010	17.4 ± 0.5	17.8 ± 0.6	0.23 ± 0.01	142 ± 10	7 ± 1	93 ± 2

S9. Distribution of the shape anisotropy constants by implementing size intervals of samples CF7 to CF9

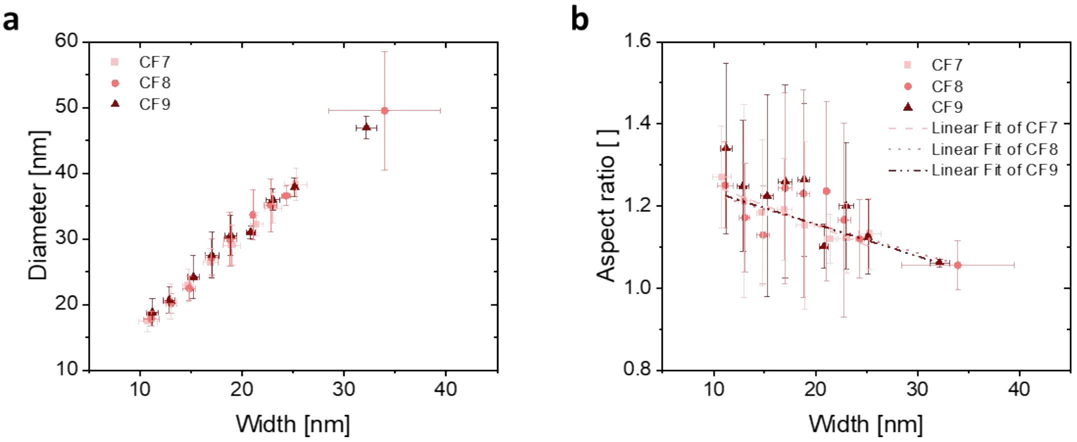

Fig. S19. Interval representation of the size and aspect ratio for width ranges of 2 nm. In dependence on the width, (**a**) the diameters increase, and (**b**) the aspect ratios decrease.

S10. Determination of the surface anisotropy constant

The response of a particle's magnetic moments to an external magnetic field influences the slope of the hysteresis curve in its linear region, described Fby the equation $M = \chi_P \cdot H$.[S7] A steeper slope indicates a higher static magnetic susceptibility, χ_P, which can be extracted from the plot of dM/dH at $H = 0$. The curves are depicted in Fig. S20.

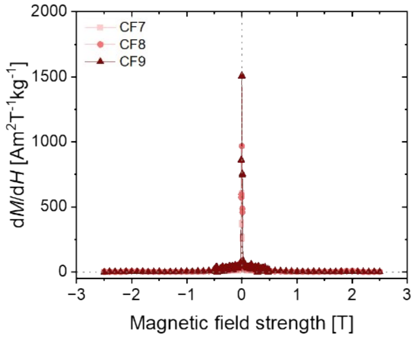

Fig. S20. Differential magnetisation curves of the samples CF7 to CF9.

It is evident that χ_P is also affected by particle size and the Co/Fe ratio. Furthermore, the magnetic domain size, d_{mag}, can be deduced by considering the condition $dM/dH_{H=0}$. Then, d_{mag} can be obtained by Eq. S9.[S8]

$$d_{mag} = \sqrt[3]{\frac{18 \cdot k_B T}{\pi \cdot \rho \cdot M_S^2} \cdot \left(\frac{dM}{dH}\right)_{H=0}} \tag{S9}$$

The spatially resolved shape anisotropy constant can be calculated *via* Eq. S10, assuming K to be nearby K_C:[S9,S10]

$K_O = K \cdot d_{mag}/6$ (S10)

S11. Additional references

S1 J. Gao, Y. Wang and H. Hao, *Front. Chem. Sci. Eng.*, 2012, **6**, 276–281.

S2 E. C. Stoner and E. P. Wohlfarth, *Philos. Trans. R. Soc. London. Ser. A, Math. Phys. Sci.,* 1948,**40**, 599–642.

S3. D. Eberbeck, *et al.*, *J. Magn. Magn. Mater.,* 2023, **583**, 171031.

S4. K. Kodama, *J. Geophys. Res. Solid Earth,* 2013, **118**, 1–12.

S5. P. C.Fannin, C. N. Marin, and Couper, C. *J. Magn. Magn. Mater.,* 2010, **322**, 1682–1685.

S6. D. Eberbeck, *Eur. Phys. J. B,* 1999, **10**, 237-245.

S7 R. K. Kotnala and J. Shah, *Handbook of Magnetic Materials: Chapter 4 – Ferrite Materials*, Elsevier, 2015, Vol. **23**, pp. 291–379.

S8 A. Aftabi, A. Babakhani and R. Khoshlahni, *Sci. Rep.,* 2025, **15**, 10039.

S9 D. Zákutná, D. Nižňanský, L. C. Barnsley, E. Babcock, Z. Salhi, A. Feoktystov, D. Honecker and S. Disch, *Phys. Rev. X*, 2020, **10**, 031019.

S10 M. Gerina, M. Sanna Angotzi, V. Mameli, M. Mazur, N. Rusta, E. Balica, P. Hrubovcak, C. Cannas, D. Honecker and D. Zákutná, *ACS Appl. Nano Mater.*, 2024, **7**, 27210–27216.
